# Supplementary material for: Distinct gene expression by expanded clones of quiescent memory CD4+ T cells harboring intact latent HIV-1 proviruses
Source: Cell Rep. 2022 Sep 6;40(10):111311. doi: 10.1016/j.celrep.2022.111311 (PMC9471989; doi:10.1016/j.celrep.2022.111311)
Supplement: Document S1. Tables S1–S9 and Figures S1–S3 [file mmc1.pdf]

Cell Reports, Volume *40*

Supplemental information

Distinct gene expression by expanded clones of quiescent memory CD4^{+} T cells harboring intact latent HIV-1 proviruses

Georg H.J. Weymar, Yotam Bar-On, Thiago Y. Oliveira, Christian Gaebler, Victor Ramos, Harald Hartweger, Gaëlle Breton, Marina Caskey, Lillian B. Cohn, Mila Jankovic, and Michel C. Nussenzweig

ID	Age	Sex	Race	Year HIV-1 Dx	Year ART initiation	Uninterr. ART (years)	Viral load (copies/mL)	$CD4^+$ T cell count	Reported nadir	ART regimen
**B207**	47	M	White/Hispanic	10	10	9	<20	724	50	EFV/TDF/FTC
**603**	43	M	White/Hispanic	12	10	10	<20	300	693	EFV/TDF/FTC
**605**	36	M	White/Hispanic	15	14	2	<20	372	524	RPV/TDF/FTC
**5104**	35	M	Black	7	7	7	<20	400	606	BIC/TAF/FTC
**5125**	34	M	Black	11	10	2	<20	450	1006	DTG/TDF/FTC
**9247**	31	M	Black	6	6	6	<20	728	600	EVG/Cobi/TAF/FTC

Table S1: Clinical characteristics of study participants. Related to STAR methods – Participant cohort.
Dx: diagnosis; Uninterr: uninterrupted; ART: antiretroviral treatment; EVG: elvitegravir; Cobi: cobicistat; TDF: tenofovir disoproxil fumarate; FTC: emtricitabine; RPV: rilpivirine; TAF: tenofovir alafenamide fumurate; BIC: bictegravir.

	603	605	B207	5104	5125	9247
**CUPM Q4PCR**	ND	ND	ND	210[1]	28[1]	15[2]
**CUPM Env PCR**	13	431	164	ND	ND	ND
**Latent clone inducible *in vitro***	Yes[3]	Yes[3]	Yes[3,4]	Yes[4]	ND	No
**Integration site**	ZNF486[4]	ND	ZPF30[4]	ATP2B4, DUXL6[4]	ND	ND

Table S2: Frequency, inducibility, and integration site of the clone of interest in CD4$^+$ T cells. Related to STAR methods – Participant cohort.
CUPM: Clonal units of clone of interest per million CD4$^+$ T cells.
[1]Gaebler (2022) [2]Gaebler (2021) [3]Cohn (2018) [4]Huang (2021)

	CD45RA	TRBC	TRBV	combined
**603**	1.5	2.2	14.3	47.2
**605**	~2 (estimate)	-	20	40
**B207**	1.6	2	2.9	9.3
**5104**	2.4	1.6	2.9	11.1
**5125**	1.5	1.8	20	54
**9247**	1.9	5	71	674.5

Table S3: Relative enrichment per marker based on flow cytometry. Related to Figure 3 and Figure S2.

	Latent clone *env* copies per 10^4 CD4$^+$ T cells	Specific TCR per 10^4 CD4$^+$ T cells	Ratio TCR/*env*
**603**	20	45	2.3
**605**	163	502	3.1
**B207**	6	14	2.3
**5104**	14	16	1.1
**5125**	15	17	1.1
**9247**	18	23	1.3

Table S4: Comparison between *env* copies per 10^4 CD4$^+$ T cells (based on Env PCR) and the frequency of the latent clone TCR (based on 10x Genomics TCR sequencing) after enrichment based on CD45RA, TRBV, and TRBC. Related to Figure 3 and Figure 4.

	5104		
	**10x TCR clonotype**	**Env^{+} (n=7)**	**Env^{-} (n=36)**
1	clonotype10133	100.0	0.0
2	clonotype66526	28.6	33.3
3	clonotype1969	14.3	11.1
4	clonotype66656	14.3	8.3
5	clonotype80542	14.3	13.9
6	clonotype19143	0.0	5.6
	**5125**		
	**clonotype**	**Env^{+} (n=4)**	**Env^{-} (n=31)**
1	clonotype91937	100.0	0.0
2	clonotype11134	25.0	3.3
3	clonotype44083	0.0	3.3
4	clonotype64266	0.0	3.3
5	clonotype89427	0.0	3.3
6	clonotype10571	0.0	0.0
	**9247**		
	**clonotype**	**Env^{+} (n=9)**	**Env^{-} (n=42)**
1	clonotype87647	88.9	0.0
3	clonotype5053	0.0	3.0
4	clonotype28375	0.0	24.2
5	clonotype72048	11.1	21.2
6	clonotype62633	11.1	18.2
7	clonotype62638	11.1	18.2
8	clonotype27093	11.1	0.0
9	clonotype46171	11.1	6.1
10	clonotype556	11.1	0.0
11	clonotype93203	11.1	0.0

Table S5: Identification of the latent clone TCR in individuals 5104, 5125, and 9247. Related to Figure 4.

Frequency of the most frequent 10x TCR clonotypes in Env^{+} and Env^{-} wells for individuals 5104, 5125, and 9247 shows a clear enrichment of only one TCR clonotype in Env^{+} wells. This TCR clonotype was designated as the latent clone TCR.

Number of cells analyzed by 10x genomics single cell gene expression							
**Individual**	**603**	**605**	**B207**	**5104**	**5125**	**9247**	**Sum**
**# of cells**	16,224	17,357	27,472	21,143	12,173	14,848	109,217

**Individual**	**603**			**605**			**B207**			**5104**			**5125**			**9247**		
**Clone**	smaller (n=64)	latent (n=75)	bigger (n=84)	smaller (n=330)	latent (n=844)	bigger	smaller (n=40)	latent (n=41)	bigger (n=42)	smaller (n=32)	latent (n=35)	bigger (n=38)	smaller (n=21)	latent (n=21)	bigger (n=22)	smaller (n=34)	latent (n=34)	bigger (n=35)
**Cluster 0**	17.2	18.7	34.5	8.5	7.8	-	0.0	12.2	35.7	43.8	17.1	0.0	33.3	9.5	9.1	8.8	0.0	0.0
**Cluster 1**	54.7	0.0	4.8	68.2	2.7	-	5.0	0.0	26.2	0.0	5.7	0.0	19.0	14.3	36.4	2.9	2.9	5.7
**Cluster 2**	1.6	0.0	0.0	0.0	0.0	-	0.0	0.0	11.9	40.6	8.6	0.0	28.6	4.8	27.3	20.6	23.5	34.3
**Cluster 3**	1.6	0.0	0.0	1.5	0.6	-	5.0	4.9	4.8	3.1	0.0	0.0	9.5	0.0	9.1	0.0	0.0	0.0
**Cluster 4**	3.1	2.7	7.1	4.5	1.2	-	25.0	2.4	0.0	3.1	2.9	0.0	0.0	4.8	13.6	35.3	8.8	31.4
**Cluster 5**	4.7	10.7	10.7	11.5	1.7	-	0.0	0.0	4.8	6.3	0.0	0.0	4.8	4.8	0.0	5.9	0.0	5.7
**Cluster 6**	4.7	0.0	0.0	0.0	0.7	-	0.0	0.0	2.4	0.0	0.0	0.0	4.8	0.0	4.5	23.5	35.3	22.9
**Cluster 7**	0.0	60.0	23.8	2.1	63.7	-	0.0	73.2	4.8	3.1	65.7	0.0	0.0	47.6	0.0	0.0	29.4	0.0
**Cluster 8**	0.0	8.0	0.0	0.0	3.8	-	0.0	7.3	0.0	0.0	0.0	100.0	0.0	9.5	0.0	0.0	0.0	0.0
**Cluster 9**	0.0	0.0	0.0	0.0	0.5	-	2.5	0.0	0.0	0.0	0.0	0.0	0.0	0.0	0.0	0.0	0.0	0.0
**Cluster 10**	0.0	0.0	0.0	0.3	0.2	-	62.5	0.0	0.0	0.0	0.0	0.0	0.0	0.0	0.0	0.0	0.0	0.0
**Cluster 11**	7.8	0.0	16.7	1.8	16.1	-	0.0	0.0	9.5	0.0	0.0	0.0	0.0	4.8	0.0	0.0	0.0	0.0
**Cluster 12**	0.0	0.0	2.4	0.0	0.4	-	0.0	0.0	0.0	0.0	0.0	0.0	0.0	0.0	0.0	2.9	0.0	0.0
**Cluster 13**	4.7	0.0	0.0	0.6	0.2	-	0.0	0.0	0.0	0.0	0.0	0.0	0.0	0.0	0.0	0.0	0.0	0.0
**Cluster 14**	0.0	0.0	0.0	0.9	0.4	-	0.0	0.0	0.0	0.0	0.0	0.0	0.0	0.0	0.0	0.0	0.0	0.0
**Sum**	100	100	100	100	100	-	100	100	100	100	100	100	100	100	100	100	100	100

Table S6: Upper part: Number of cells analyzed by 10x Genomics per individual. Lower part: Distribution [%] of the latent clone, the next smaller, and the next bigger clone per individual over the 15 gene expression clusters. Related to Figure 5.

Individual	Cluster 7					
	# of cells	# of latent cells	# [%] of other cells	# [%] of other cells in clones	# of other clones	# [%] of singlet T cells
603	887	45	842 [94.9]	581 [65.5]	330	261 [29.4]
605	1,989	538	1,451 [73]	1,156 [58.1]	498	295 [14.8]
B207	1,295	30	1,265 [97.7]	890 [68.7]	526	375 [29]
5104	960	23	937 [97.6]	655 [68.2]	383	282 [29.4]
5125	297	10	287 [96.6]	223 [75.1]	111	64 [21.5]
9247	358	10	348 [97.2]	318 [88.8]	100	30 [8.4]

Table S7: Contribution of non-latent clones and single cells to cluster 7. Related to Figure 5.

Ensembl gene ID	External gene name	p value	Average log2 fold change
ENSG00000115687	PASK	2.5074E-273	-0.937004582
ENSG00000188404	SELL	1.1432E-303	-0.757375402
ENSG00000168209	DDIT4	4.2236E-135	-0.753863566
ENSG00000070756	PABPC1	1.3615E-275	-0.577338107
ENSG00000074800	ENO1	3.9911E-72	-0.552495574
ENSG00000273149	AL138963.4	1.69448E-43	-0.503468146
ENSG00000111669	TPI1	3.75841E-56	-0.502939955
ENSG00000095794	CREM	7.5629E-48	-0.48733596
ENSG00000157601	MX1	3.44639E-38	-0.463685933
ENSG00000149212	SESN3	2.09806E-96	-0.458160858
ENSG00000126353	CCR7	5.6185E-113	-0.438022627
ENSG00000177410	ZFAS1	9.1716E-145	-0.432744809
ENSG00000173762	CD7	1.2733E-119	-0.422179842
ENSG00000059804	SLC2A3	4.68348E-68	-0.401753273
ENSG00000197061	HIST1H4C	1.74666E-54	-0.367052221
ENSG00000234741	GAS5	1.8049E-124	-0.365481063
ENSG00000134333	LDHA	3.62429E-18	-0.356852098
ENSG00000197989	SNHG12	2.6524E-59	-0.354230806
ENSG00000105220	GPI	2.30295E-38	-0.33651571
ENSG00000146278	PNRC1	2.56242E-66	-0.33232545
ENSG00000196352	CD55	3.58249E-86	-0.331613182
ENSG00000269028	MTRNR2L12	9.28887E-42	-0.326076812
ENSG00000251562	MALAT1	4.23243E-61	-0.326073295
ENSG00000240972	MIF	1.16951E-48	-0.325935464
ENSG00000067225	PKM	3.0003E-53	-0.324450491
ENSG00000166012	TAF1D	4.1997E-62	-0.323635813
ENSG00000130066	SAT1	6.88644E-24	-0.320711104
ENSG00000161011	SQSTM1	3.25091E-23	-0.307785538
ENSG00000181163	NPM1	1.7052E-145	-0.295019474
ENSG00000081059	TCF7	2.53957E-59	-0.293559954
ENSG00000100906	NFKBIA	6.90414E-22	-0.286544704
ENSG00000138795	LEF1	4.65739E-63	-0.283635236
ENSG00000102144	PGK1	3.47758E-18	-0.283588302
ENSG00000104765	BNIP3L	4.68824E-42	-0.280401542
ENSG00000144381	HSPD1	1.33312E-51	-0.27754675
ENSG00000096384	HSP90AB1	6.19504E-51	-0.27513593
ENSG00000114023	FAM162A	3.98958E-38	-0.271026449
ENSG00000167658	EEF2	2.0542E-116	-0.263184273
ENSG00000081320	STK17B	1.01417E-40	-0.263109056
ENSG00000105193	RPS16	7.12703E-82	-0.25357902

ENSG00000213145	CRIP1	1.84831E-74	0.251381711
ENSG00000204642	HLA-F	1.67539E-32	0.252987681
ENSG00000064666	CNN2	7.14478E-34	0.254603703
ENSG00000136167	LCP1	4.88255E-38	0.255115632
ENSG00000145247	OCIAD2	1.35036E-33	0.255136956
ENSG00000135441	BLOC1S1	8.55057E-29	0.256708541
ENSG00000217555	CKLF	6.26258E-37	0.268552743
ENSG00000179218	CALR	5.15079E-26	0.269118004
ENSG00000166710	B2M	0	0.270596986
ENSG00000034713	GABARAPL2	7.97742E-47	0.271789708
ENSG00000213626	LBH	1.70427E-35	0.275234928
ENSG00000158062	UBXN11	2.96782E-31	0.277861974
ENSG00000135046	ANXA1	6.7754E-104	0.277916253
ENSG00000240065	PSMB9	9.1741E-63	0.278633877
ENSG00000165929	TC2N	3.38931E-34	0.282431558
ENSG00000136810	TXN	4.66479E-33	0.284410494
ENSG00000115232	ITGA4	4.07453E-43	0.296034223
ENSG00000234745	HLA-B	2.442E-301	0.299434559
ENSG00000182718	ANXA2	1.2129E-48	0.300086815
ENSG00000105404	RABAC1	3.41798E-59	0.300856113
ENSG00000108518	PFN1	5.1347E-192	0.308425928
ENSG00000075624	ACTB	3.1245E-173	0.312823868
ENSG00000163191	S100A11	5.8168E-123	0.313585328
ENSG00000213719	CLIC1	3.2501E-80	0.321146717
ENSG00000126246	IGFLR1	1.12795E-32	0.321234367
ENSG00000027869	SH2D2A	1.2519E-59	0.325040074
ENSG00000008517	IL32	1.885E-203	0.326877861
ENSG00000100300	TSPO	4.23815E-58	0.328869744
ENSG00000130592	LSP1	5.13406E-85	0.335795569
ENSG00000170571	EMB	1.30243E-48	0.340520299
ENSG00000110324	IL10RA	7.30307E-71	0.345878196
ENSG00000092841	MYL6	1.8186E-212	0.349021829
ENSG00000160255	ITGB2	1.63101E-55	0.376681806
ENSG00000197747	S100A10	5.2906E-220	0.381136641
ENSG00000169442	CD52	2.9441E-231	0.386540002
ENSG00000132965	ALOX5AP	2.30411E-89	0.390330172
ENSG00000148362	PAXX	1.9717E-94	0.391613282
ENSG00000197540	GZMM	5.469E-80	0.411312119
ENSG00000002586	CD99	1.1703E-191	0.411377898
ENSG00000111796	KLRB1	8.73173E-56	0.434616046
ENSG00000196154	S100A4	9.403E-205	0.446707794

ENSG00000142669	SH3BGRL3	0	0.473220735
ENSG00000126264	HCST	3.4505E-135	0.484457968
ENSG00000103187	COTL1	8.1033E-136	0.491151274
ENSG00000100097	LGALS1	3.0001E-118	0.520481509
ENSG00000116824	CD2	7.0921E-219	0.527921045
ENSG00000051523	CYBA	0	0.535424541
ENSG00000133321	PLAAT4	1.0403E-293	0.6130956
ENSG00000235576	LINC01871	6.8684E-252	0.712737658
ENSG00000186810	CXCR3	0	0.863806843
ENSG00000145220	LYAR	0	0.91690397
ENSG00000223865	HLA-DPB1	0	1.005834612
ENSG00000077984	CST7	0	1.00668931
ENSG00000231389	HLA-DPA1	0	1.0517126
ENSG00000145649	GZMA	0	1.159266639
ENSG00000196126	HLA-DRB1	0	1.189048765
ENSG00000158050	DUSP2	0	1.377812382
ENSG00000271503	CCL5	0	1.450021207
ENSG00000019582	CD74	0	1.478166065
ENSG00000113088	GZMK	0	2.055910352

Table S8: Differentially expressed genes (average log2fold < -2.5; average log2fold > 2.5) in cluster 7 compared to all other clusters. Related to Figure 6.

	603	605	B207	5104	5125	9247
**CD4 CTL**	1.6%	58.5%	0.0%	0.0%	0.9%	0.0%
**CD4 Naïve**	0.0%	1.0%	0.0%	0.0%	0.0%	0.0%
**CD4 Proliferating**	0.0%	0.0%	0.0%	0.0%	0.0%	0.0%
**CD4 TCM**	0.1%	2.2%	0.0%	0.1%	0.1%	0.2%
**CD4 TEM**	6.1%	21.9%	2.4%	1.1%	3.8%	2.0%
**Treg**	0.0%	0.4%	0.0%	0.0%	0.0%	0.0%

Table S9: Percentage of cell pertaining to the latent clone relative to cluster size. Related to Figure 7.

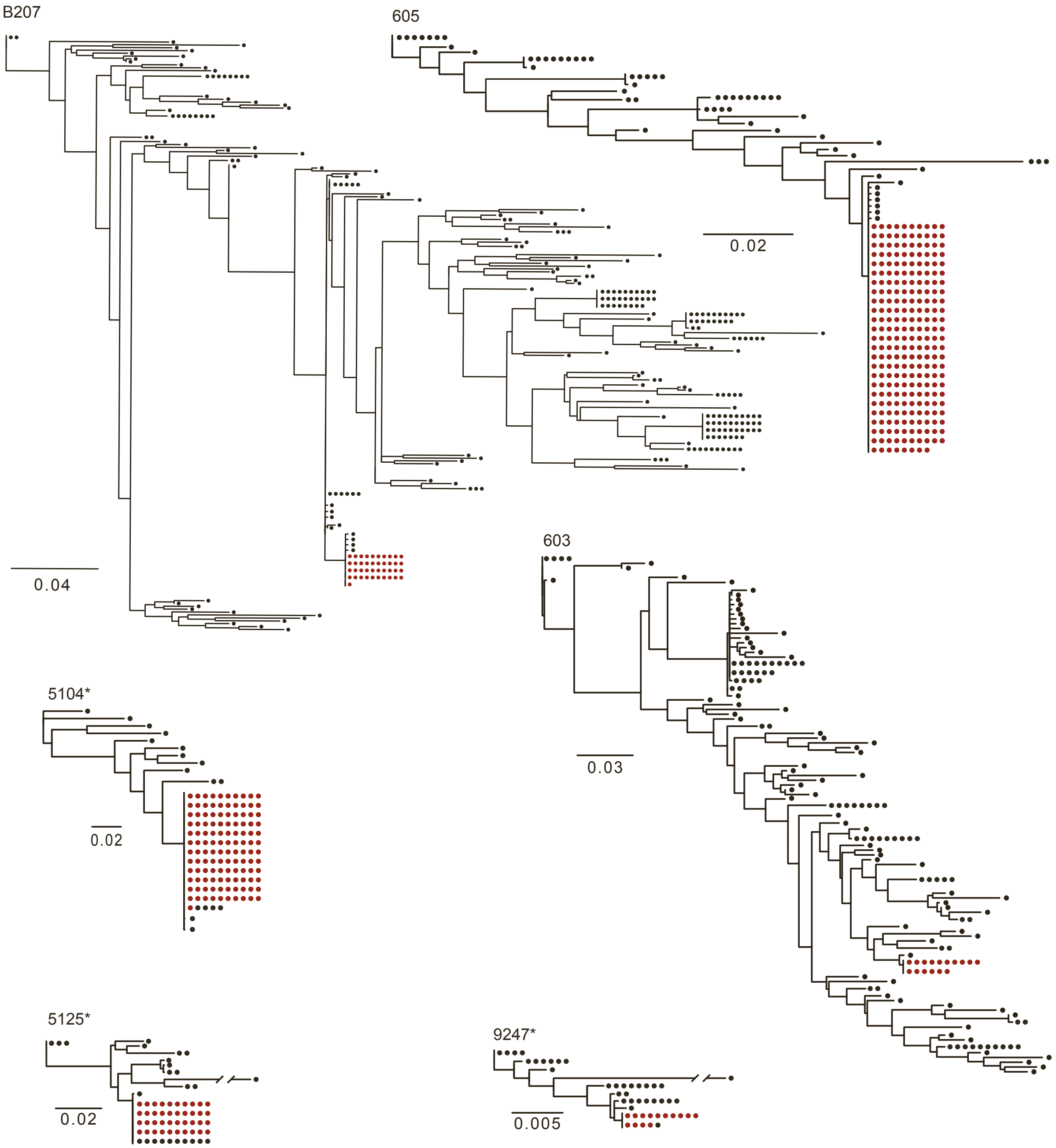

Figure S1 The latent reservoir in each individual contains an expanded intact latent clone. Related to STAR methods - Participant cohort.

Maximum-likelihood phylogenetic trees show the *env* gene of the clone of interest marked in red. The scale bars indicate the number of substitutions per site. Each dot on the maximum-likelihood phylogenetic trees represents an *env* sequence that was recovered by Env PCR (individuals B207, 603, and 605) or Q4PCR (marked with asterisk; individuals 5104, 5125, and 9247 (Gaebler et al., 2021; Gaebler et al., 2022)) from CD4^{+} T cells before enrichment. For individuals 603, 605, and B207 the *env* gene was also found in viral outgrowth assays (Cohn et al., 2018).

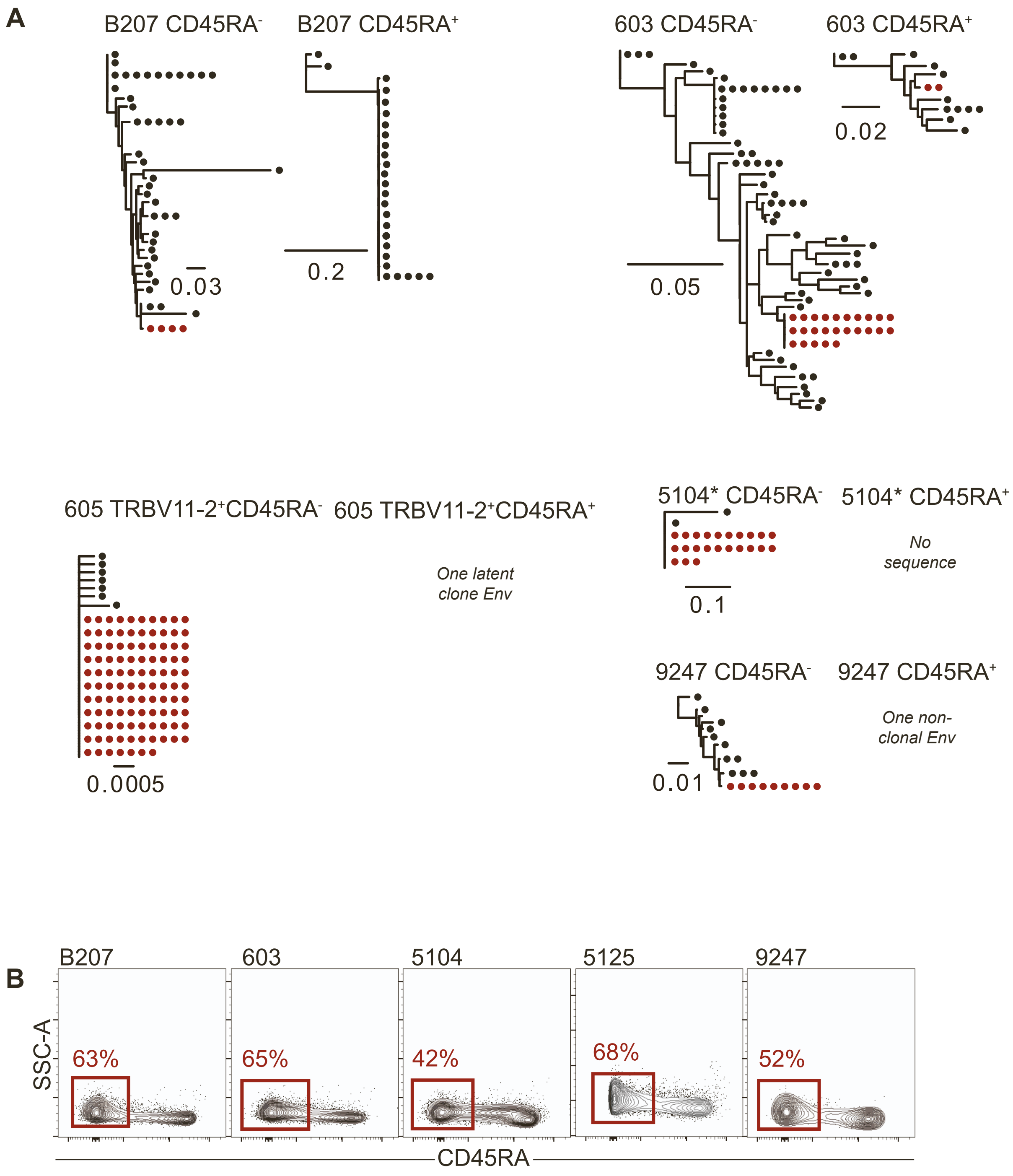

Figure S2 The latent clone of interest is enriched in the CD45RA- compartment. Related to Figure 1 and Table S3.
Maximum-likelihood phylogenetic trees show *env* gene of the clone of interest marked in red. The scale bars indicate the number of substitutions per site. Asterisks indicate trees based on *env* sequences obtained from Q4PCR (Gaebler et al., 2019), all other trees are based on Env PCR. Each sort was performed once.
(A) Magnetic negative selection of CD45RA- memory $CD4^+$ T cells enriches the latent clone of interest in all 6 individuals.
(B) Flow cytometry plots indicating the fraction of CD45RA- cells among $CD4^+$ T cells.

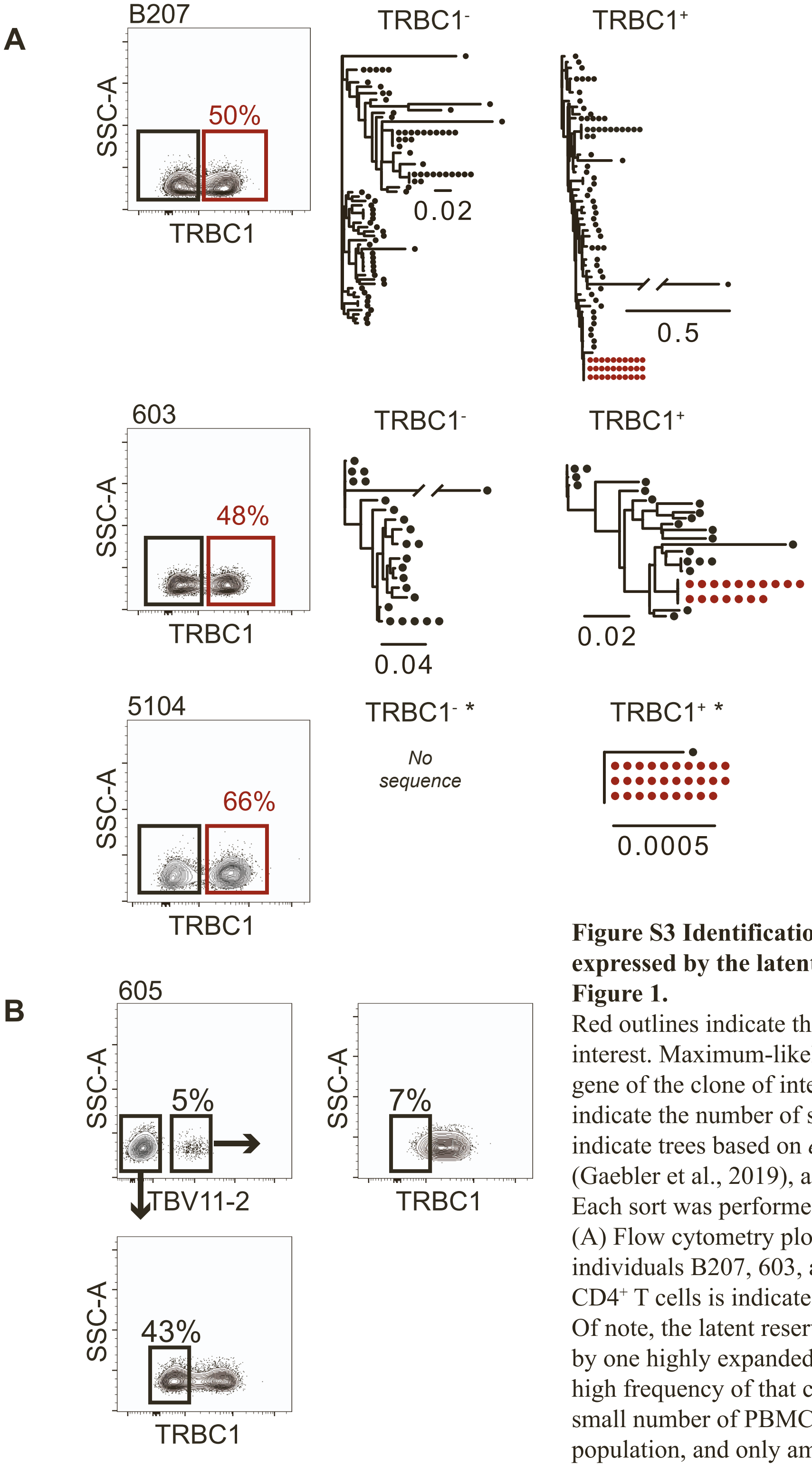

Figure S3 Identification of the TRBC domain that is expressed by the latent clone of interest. Related to Figure 1.

Red outlines indicate the population containing the clone of interest. Maximum-likelihood phylogenetic trees show *env* gene of the clone of interest marked in red. The scale bars indicate the number of substitutions per site. Asterisks indicate trees based on *env* sequences obtained from Q4PCR (Gaebler et al., 2019), all other trees are based on Env PCR. Each sort was performed once.

(A) Flow cytometry plots show TRBC1 staining for individuals B207, 603, and 5104. The fraction of TRBC1$^+$ CD4$^+$ T cells is indicated.

Of note, the latent reservoir of individual 5104 is dominated by one highly expanded single clone (Figure S1). Due to the high frequency of that clone, we only needed to assay a small number of PBMCs to detect that clone in the TRBC1$^+$ population, and only amplified one other sequence by Q4PCR. Therefore, much less frequent latent cells that possibly were TRBC1$^-$ were not captured.

(B) Flow cytometry plots show TRBC1 staining for TRBV11-2$^-$ and TRBV11-2$^+$ cells in individual 605. The fraction cells within a gate is indicated above the gate.
