# Supplementary material for: Distinct gene expression by expanded clones of quiescent memory CD4+ T cells harboring intact latent HIV-1 proviruses
Source: Cell Rep. 2022 Sep 6;40(10):111311. doi: 10.1016/j.celrep.2022.111311 (PMC9471989; doi:10.1016/j.celrep.2022.111311)
Supplement: Document S2. Article plus supplemental information [file mmc3.pdf]

Cell Reports

Article

Distinct gene expression by expanded clones of quiescent memory CD4$^+$ T cells harboring intact latent HIV-1 proviruses

Graphical abstract

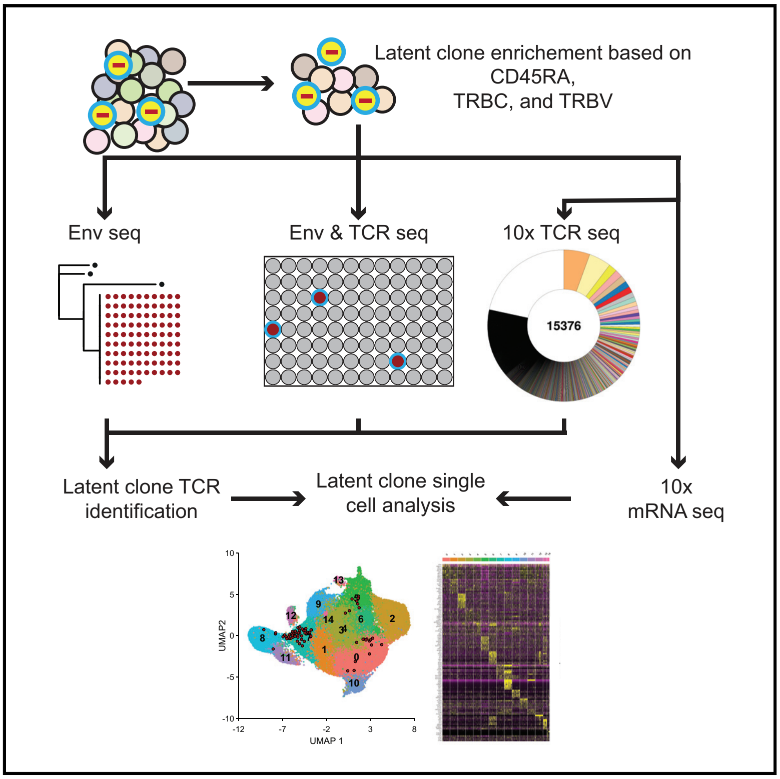

Highlights

- Intact latent clones can be enriched and identified by their TCR
- Intact latent clones display a heterogeneous gene expression profile
- Intact latent clones are enriched in the effector memory compartment

Authors

Georg H.J. Weymar, Yotam Bar-On, Thiago Y. Oliveira, ..., Lillian B. Cohn, Mila Jankovic, Michel C. Nussenzweig

Correspondence

nussen@rockefeller.edu

In brief

Weymar et al. enrich and identify rare CD4$^+$ T cell clones harboring intact HIV-1 proviruses based on their unique T cell receptor. The gene expression analysis of confirmed intact latent clones reveals that these clones display a heterogeneous gene-expression profile but are enriched in the effector memory compartment.

Weymar et al., 2022, Cell Reports *40*, 111311
September 6, 2022 © 2022 The Author(s).
https://doi.org/10.1016/j.celrep.2022.111311

CellPress

Cell Reports

CellPress
OPEN ACCESS

Article

Distinct gene expression by expanded clones of quiescent memory CD4$^+$ T cells harboring intact latent HIV-1 proviruses

Georg H.J. Weymar,[1] Yotam Bar-On,[2] Thiago Y. Oliveira,[1] Christian Gaebler,[1] Victor Ramos,[1] Harald Hartweger,[1] Gaëlle Breton,[1] Marina Caskey,[1] Lillian B. Cohn,[3] Mila Jankovic,[1] and Michel C. Nussenzweig[1,4,5,*]

[1]Laboratory of Molecular Immunology, The Rockefeller University, New York, NY 10065, USA
[2]Technion - Israel Institute of Technology, Haifa 320003, Israel
[3]Vaccine and Infectious Diseases Division, Fred Hutchinson Cancer Research Center, Seattle, WA 98109, USA
[4]Howard Hughes Medical Institute, Chevy Chase, MD 20815, USA
[5]Lead contact
*Correspondence: nussen@rockefeller.edu
https://doi.org/10.1016/j.celrep.2022.111311

SUMMARY

Antiretroviral therapy controls, but does not cure, HIV-1 infection due to a reservoir of rare CD4$^+$ T cells harboring latent proviruses. Little is known about the transcriptional program of latent cells. Here, we report a strategy to enrich clones of latent cells carrying intact, replication-competent HIV-1 proviruses from blood based on their expression of unique T cell receptors. Latent cell enrichment enabled single-cell transcriptomic analysis of 1,050 CD4$^+$ T cells belonging to expanded clones harboring intact HIV-1 proviruses from 6 different individuals. The analysis reveals that most of these cells are T effector memory cells that are enriched for expression of *HLA-DR*, *HLA-DP*, *CD74*, *CCL5*, granzymes A and K, cystatin F, *LYAR*, and *DUSP2*. We conclude that expanded clones of latent cells carrying intact HIV-1 proviruses persist preferentially in a distinct CD4$^+$ T cell population, opening possibilities for eradication.

INTRODUCTION

Antiretroviral therapy prevents HIV-1 viral replication but does not impact latent proviruses that are integrated into the genome of host CD4$^+$ T cells. The reservoir of latent proviruses is responsible for rapid rebound viremia in most individuals undergoing treatment interruption and is the key impediment to HIV-1 cure (Bachmann et al., 2019; Chun et al., 1997, 2010; Crooks et al., 2015; Finzi et al., 1999; Kearney et al., 2016; Peluso et al., 2020; Siliciano et al., 2003; Wong et al., 1997).

Although the precise composition of the latent compartment is not known, the relative representation of expanded clones of CD4$^+$ T cells harboring intact and defective latent proviruses increases over time such that they account for at least 50% of the reservoir in chronically infected individuals (Antar et al., 2020; Bui et al., 2017; Cho et al., 2022; Cohn et al., 2015; De Scheerder et al., 2019; Einkauf et al., 2022; Hosmane et al., 2017; Lorenzi et al., 2016). Members of infected clones express the same unique T cell receptor (TCR) and have a single distinctive proviral integration site, each of which can serve as a molecular identifier for the latent clone (Cohn et al., 2018; Einkauf et al., 2019, 2022; Huang et al., 2021; Simonetti et al., 2021). Hypotheses about how the latent reservoir is maintained include proviral integration sites that enable cell division (Maldarelli et al., 2014; Wagner et al., 2014) and homeostatic (Chomont et al., 2009) and antigen-driven proliferation (Douek et al., 2002; Gantner et al., 2020; Henrich et al., 2017; Mendoza et al., 2020; Simonetti et al., 2021).

Because cells harboring intact latent proviruses are rare (Bachmann et al., 2019; Crooks et al., 2015; Ho et al., 2013; Peluso et al., 2020; Sengupta and Siliciano, 2018) and have no well-defined markers that distinguish them from other CD4$^+$ T cells (Cohn et al., 2020; Darcis et al., 2019), characterizing their transcriptional program has not been possible to date. Intact proviruses are enriched among CD4$^+$CD45RA$^-$HLA-DR$^+$ memory T cells (Chomont et al., 2009; Cockerham et al., 2014; Hiener et al., 2017; Horsburgh et al., 2020; Lee et al., 2017, 2019), but other surface markers, such as CD2 (Iglesias-Ussel et al., 2013), remain controversial. Latent cells can be identified after re-activation of HIV-1 transcription *in vitro* (Baxter et al., 2016; Cohn et al., 2018; Grau-Exposito et al., 2019; Liu et al., 2020; Neidleman et al., 2020; Pardons et al., 2019), and there are numerous cell line- or tissue culture-based models of latency (Cameron et al., 2010; Iglesias-Ussel et al., 2013; Krishnan and Zeichner, 2004; Lassen et al., 2012; Marini et al., 2008; Telwatte et al., 2019), but how well these experimental conditions and latency models reflect the physiology of latent cells in circulation is not known.

Here, we present a strategy to enrich clones of quiescent latent cells from samples that were assayed directly *ex vivo* from 6 individuals living with HIV-1 based on cell-surface expression of their unique TCRs. The enrichment strategy enabled analysis of the transcriptional landscape of these rare cells and identification of distinct features of this population.

This is an open access article under the CC BY license (http://creativecommons.org/licenses/by/4.0/).

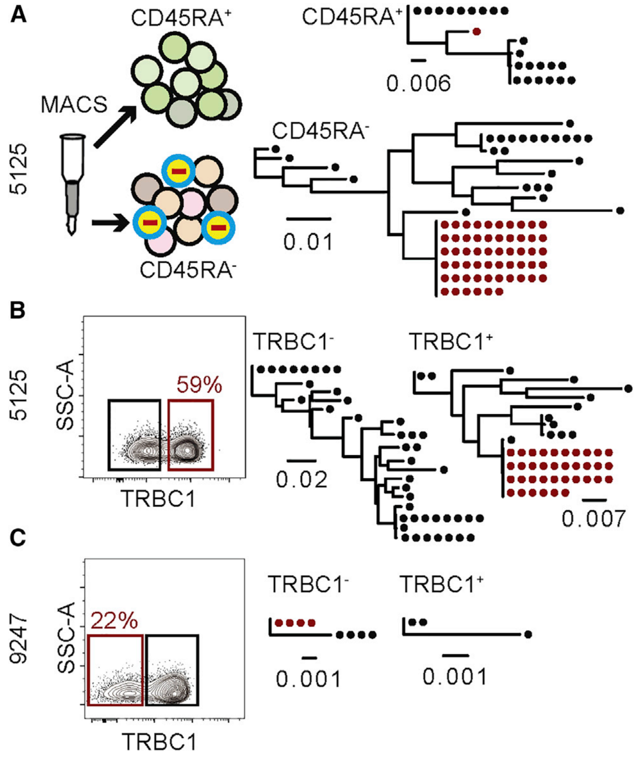

Figure 1. Screening for intact latently infected cell enrichment by sorting for CD45RA and TRBC

Red outlines indicate the population containing the clone of interest. Maximum-likelihood phylogenetic trees show *env* gene of the latent clone of interest marked in red. Each dot represents a recovered *env* sequence from the respective subpopulation of CD4$^+$ T cells. The scale bars indicate the number of substitutions per site. Each sort was performed once. See also Figures S2 and S3 and Table S3.

(A) CD4$^+$ T cells from individual 5125 were magnetically sorted into CD45RA$^+$ and CD45RA$^-$ populations. Subsequent gDNA extraction, limiting dilution, and *env* sequencing revealed that the infected clone of interest in was enriched in the CD45RA$^-$ memory compartment.

(B) CD4$^+$ T cells from individual 5125 were stained with anti-TRBC1 antibody and sorted into TRBC1$^+$ and TRBC1$^-$ populations. The latent clone was enriched in the TRBC1$^+$ compartment.

(C) CD4$^+$ T cells from individual 9247 were stained with anti-TRBC1 antibody and sorted into TRBC1$^+$ and TRBC1$^-$ populations. The latent clone was enriched in the TRBC1$^-$ compartment.

RESULTS

During development, T and B lymphocytes assemble unique cell-surface receptors by variable, diversity, and joining gene (V(D)J) recombination. This process is under feedback regulation by the receptor such that each lymphocyte expresses a single specificity, a phenomenon referred to as allelic exclusion (Nussenzweig et al., 1987). Because antigen receptors are fixed early in development, naive T cells that become activated and expand produce clones of CD4$^+$ T cells that are defined by expression of a singular TCR. Thus, the TCR expressed by a clone of latent cells is a unique molecular identifier for members of that clone, and because it is a cell-surface protein, the TCR can be used to enrich members of the latent clone.

People living with HIV-1 frequently harbor large, expanded clones of latent CD4$^+$ T cells. We studied 6 chronically infected individuals controlled on antiretroviral therapy that were aviremic at the time of sample collection (Table S1). Near-full-length sequencing, envelope (*env*) gene sequencing, and/or viral outgrowth assays showed that the latent reservoir of each of these individuals was dominated by a single expanded intact latent proviral clone (Table S2) (Cohn et al., 2018; Gaebler et al., 2019, 2021, 2022; Huang et al., 2021; Lorenzi et al., 2016; Mendoza et al., 2020). In all cases, the members of these clones could be identified by the sequence of their *env* gene (Figure S1). The frequency of the clonally expanded latent provirus of interest in the 6 individuals ranged from 13–431/10^6 total CD4$^+$ T cells based on the frequency of the specific HIV-1 *env* sequence (Table S2).

Latent cells are predominantly found in the CD45RA$^-$ memory T cell compartment (Chomont et al., 2009; Hiener et al., 2017; Morcilla et al., 2021). To determine whether the CD4$^+$ T cells harboring latent proviral clones of interest are in the memory compartment, we purified CD45RA$^+$ and CD45RA$^-$ cells and analyzed proviral DNA by sequencing the *env* gene and producing maximum-likelihood phylogenetic trees. As expected, in all cases, the latent clone of interest was predominantly found in the CD45RA$^-$ population (Figures 1A and S2A). Based on staining with an anti-CD45RA antibody and flow cytometric analysis of CD4$^+$ T cells from five individuals, CD45RA$^-$ cells accounted for 42%–68% of all CD4$^+$ T cells and therefore purification of CD45RA$^-$ cells results in a 1.5- to 2.4-fold enrichment of the latent clone (Figure S2B).

The TCRβ locus encodes 2 different constant region genes (*TRBC1* and *TRBC2*). Allelic exclusion ensures that all members of a CD4$^+$ T cell clone express the same TCRβ constant region. To determine which of the 2 different TCRβ constant regions is expressed by each of the latent clones of interest, we performed flow cytometry experiments to purify TRBC1$^+$ and TRBC1$^-$ CD4$^+$ T cells. In each case, *env* sequencing revealed that the latent provirus was found among CD4$^+$ T cells expressing one of the two TRBC domains, resulting in 1.5- to 4.5-fold enrichment (Figures 1B, 1C, and S3).

The TCRβ locus contains 48 functional variable domains (*TRBV*). To determine whether the TRBV can be used to enrich quiescent clones of latent cells, we made use of a collection of 24 different anti-TRBV monoclonal antibodies. The antibodies were divided into 8 groups of 3 each that were conjugated with either phycoerythrin (PE), fluorescein isothiocyanate (FITC), or both (PEFITC).

The *TRBV* expressed by CD4$^+$ T cells harboring the latent HIV-1 proviral clones of interest in individuals 603, 605, and B207 were known (*TRBV-19*, *TRBV11-2*, and *TRBV7-8* respectively) (Cohn et al., 2018). However, the TRBV expressed by CD4$^+$ T cells harboring the latent HIV-1 proviral clones of interest in individuals 5104, 5125, and 9247 were not known. To identify the *TRBV* expressed by CD4$^+$ T cells that harbor the latent clone in these individuals, we combined limiting dilution cell sorting with *env* sequencing. As a first screening step, CD4$^+$ T cells were stained with the 24 anti-TRBV antibodies and sorted into TRBV$^+$ and TRBV$^-$ populations. The latent clone in individual 5104 was present in the TRBV$^-$ population, yielding a 2-fold

enrichment (Figure 2A). For individuals 5125 and 9247, the latent clone was found in the TRBV$^+$ population (Figures 2B and 2C). To identify the precise *TRBV* expressed by the latent clone in individual 9247, the 24 anti-TRBV antibodies were split into 2 groups of 12 antibodies, and CD4$^+$ T cells were stained with either one of the 2 groups (Figure 2D). The latent clone was found in one of the 2 groups, and the 12 antibodies split again into 4 groups of 3 anti-TRBV antibodies, with each group containing one FITC-, one PE-, and one PEFITC-labeled antibody (Figure 2E). The latent clone was only found in one of the 4 groups, and the 3 anti-TRBV antibodies in that group were then used to stain and sort for each of the anti-TRBVs individually (Figure 2F). These experiments revealed that in individual 9247, the latent clone expresses *TRBV4-3* (Figure 2F). For individual 5125, the 24 TRBV antibodies were split into 8 groups of 3, and the latent clone was found in only one of the groups (Figure 2G). The 3 antibodies in that group were then used to purify the individual TRBV-expressing cells, which showed that the latent clone of interest was found among TRBV2$^+$ cells (Figure 2H).

To determine the amount of enrichment that could be achieved for each individual, we combined anti-CD45RA, -TRBC, and -TRBV enrichment and performed limiting dilution, *env* amplification, and sequencing on genomic DNA (Figure 3; Table S3).

To enrich the latent clone in individual 603, CD4$^+$ T cells were magnetically sorted for CD45RA$^-$ cells and then stained with antibodies to TRBC1 and TRBV19. The latent clone was found in the CD45RA$^-$TRBC1$^+$TRBV19$^+$ population, resulting in an overall 47-fold relative enrichment (Figure 3A; Table S3). The latent clone in individual 605 was enriched 40-fold in the CD45RA$^-$ TRBV11-2$^+$ population (Figures 3B and S3B; Table S3). Antibodies to TRBV7-8 expressed by the latent clone in individual B207 were not available. Therefore, B207 CD4$^+$ T cells were stained with the 24 anti-TRBV antibodies and sorted into a CD45RA$^-$TRBC1$^+$TRBV$^-$ population, resulting in only a 9-fold overall enrichment (Figure 3C; Table S3). The latent clone in individual 5104 was found in the CD45RA$^-$TRBC1$^+$TRBV$^-$ population with an 11-fold enrichment (Figure 3D; Table S3). The latent clone in individual 5125 was found in the CD45RA$^-$TRBC1$^+$TRBV2$^+$ population with a 54-fold enrichment (Figure 3E; Table S3). Lastly, the latent clone in individual 9247 was found in the CD45RA$^-$TRBC1$^-$TRBV4-3$^+$ population with a 675-fold enrichment (Figure 3F; Table S3).

After combined enrichment, the latent provirus of interest was found in 20, 163, 6, 14, 15, and 18 in 10^4 CD4$^+$ T cells in individuals 603, 605, B207, 5104, 5125, and 9247, respectively (Table S4).

Although we were able to identify the *TRBC* and *TRBV* expressed by expanded clones of interest in individuals 5104, 5125, and 9247, the precise TCR$\alpha\beta$ sequence remained unknown. Candidate TCRs were initially identified among clones of CD4$^+$ T cells in the enriched populations by single-cell TCR sequencing by 10x Genomics (Figure 4A). To definitively determine the TCR expressed by the clone of CD4$^+$ T cells harboring the latent provirus, we combined anti-CD45RA, -TRBC, and -TRBV staining and sorted 5 candidate cells into multi-well plates, followed by DNA and RNA extraction. *Env* amplification and sequencing from genomic DNA were used to identify wells containing latent cells. cDNA from wells containing the latent provirus or negative controls was used to amplify and sequence TCRα and β chains (Figure 4A). CD4$^+$ T cells from individuals 603 and 605 that express known TCRs were used as positive controls to validate the method (Figures 4B and 4C). For individual 603, the TCR expressed by the latent clone was found in 63% of Env$^+$ wells and only in 4% of Env$^-$ wells (Figure 4B). In 605, the specific TCR was found in 90% of the Env$^+$ wells and only in 6% of Env$^-$ wells. In both cases, there was no enrichment of irrelevant TCRs in Env$^+$ wells. All 7 Env$^+$ wells obtained from individual 5104 contained TCRα *TRAV12-3/J9* and/or TCRβ *TRBV5-4/J1-1*, representing a unique TCR clone in the 10x Genomics sequencing data (Figures 4B and 4C). This TCR was absent in the random selection of Env$^-$ wells, and no other TCR was enriched in Env$^+$ wells (Table S5). Similarly, all 4 Env$^+$ wells from individual 5125 contained *TRAV26-2/J32* and/or *TRBV2/J1-1*, which was not found among Env$^-$ wells. Finally, in individual 9247, *TRAV38-1/J33* and/or *TRBV4-3/J2-3* was present in 8 out of 9 of Env$^+$ wells but absent in the random selection of Env$^-$ wells. We conclude that the latent clone of interest in 5104, 5125, and 9247 express *TRAV12-3/J9/TRBV5-4/J1-1*, *TRAV26-2/J32/TRBV2/J1-1*, and *TRAV38-1/J33/TRBV4-3/J2-3*, respectively (Figure 4C).

HIV-1 proviruses can integrate into CD4$^+$ T cells undergoing clonal expansion at the time they start dividing or sometime thereafter. Proviral integration in early stages of clonal expansion would yield a homogenous group of cells, the vast majority of which would harbor an HIV-1 provirus in the same genomic location. Integration at a later time would produce a heterogeneous CD4$^+$ T cell clone wherein only some of the cells in the expanded clone harbor the HIV-1 provirus (Simonetti et al., 2021). To estimate the fraction of infected cells within a particular clone based on its representation by TCR, we performed 10x Genomics single-cell TCR sequencing on samples enriched using the antibody methods described above. We compared the TCR frequencies with the relative frequency of the specific *env* from proviral DNA in similarly enriched samples (Table S4). For individuals 5104, 5125, and 9247, the frequency of the specific provirus was similar to the frequency of corresponding TCR, but in B207, 603, and 605, the number of cells expressing the specific TCR of interest was 2–3 times higher than the frequency of proviral copies (Table S4). Thus, there is heterogeneity among clones of expanded CD4$^+$ T cells that harbor latent HIV-1 proviruses. In half of our samples, most clonally expanded CD4$^+$ T cells harbor latent HIV-1, and in the others, the provirus is found in a fraction of the clone.

To determine whether CD4$^+$ T cell clones harboring latent proviruses share a transcriptional profile, we combined the 10x Genomics mRNA and TCR sequencing data obtained from the enriched populations of latent cells. TCR sequencing data were used to identify CD4$^+$ T cell clones harboring the latent HIV-1 provirus but were omitted for gene-expression analysis to prevent TCR-biased clustering. Uniform manifold approximation and projection (UMAP) analysis of the transcriptional profile of all 109,217 cells from the six individuals produced 15 unique clusters of CD4$^+$ memory T cells (Figure 5A). Visual inspection revealed that CD4$^+$ T cells expressing the specific TCR associated with latent proviruses are found predominantly in gene expression cluster 7 and neighboring clusters (Figure 5A). On average,

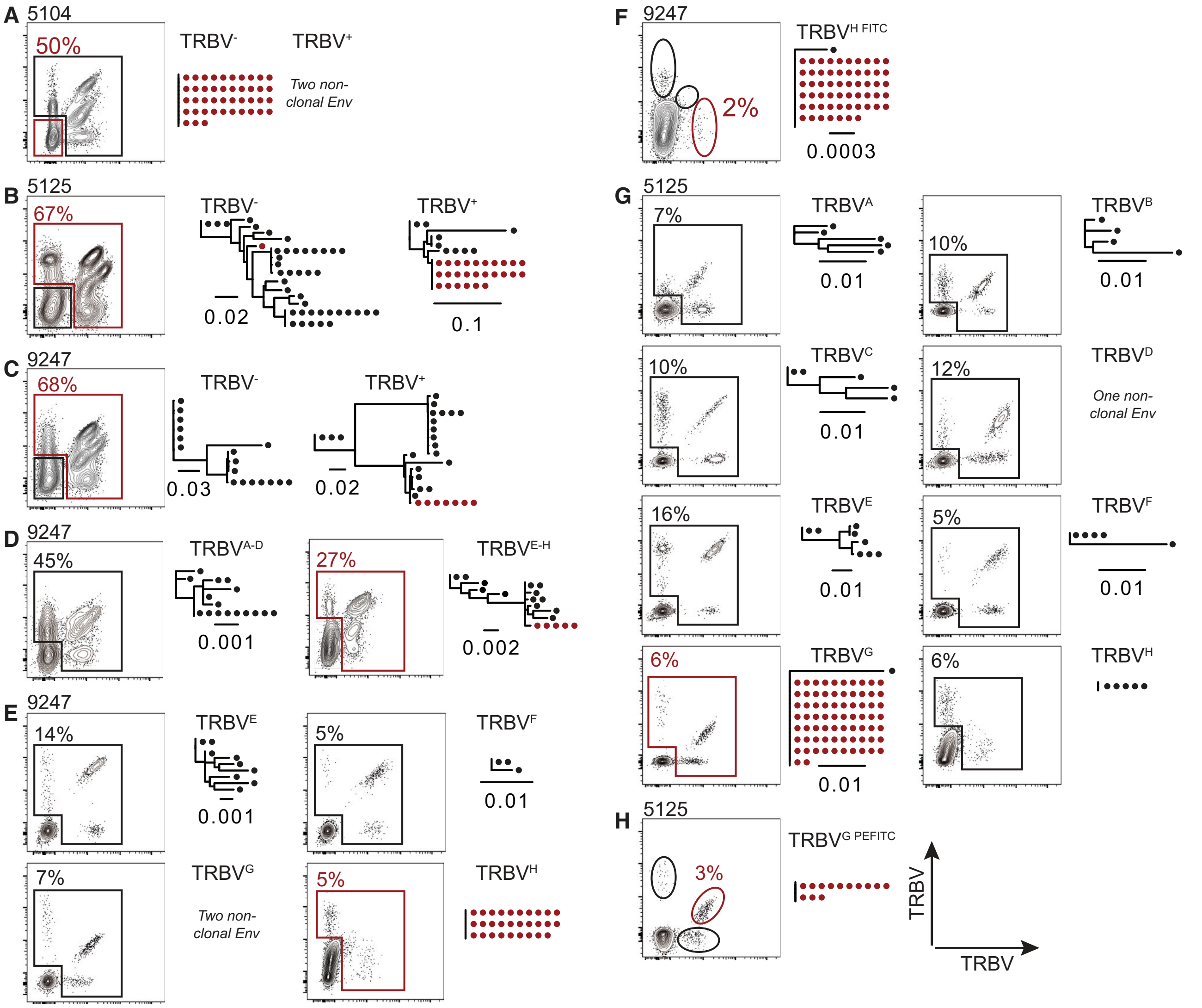

Figure 2. Screening for the TRBV expressed by the latent clone in individuals 5104, 5125, and 9247

Red outlines indicate the population containing the clone of interest. Maximum-likelihood phylogenetic trees of *env* sequences derived from sorted population, as indicated. *Env* gene of the clone of interest marked in red. The scale bars indicate the number of substitutions per site. TRBV^{A-H} indicate the grouping of monoclonal antibodies used in the cocktails (see STAR Methods).

(A–C) Flow cytometry plots show TRBV staining using a combination of 24 anti-TRBV antibodies. Percentage of TRBV$^-$ cells is indicated for individual 5104 and TRBV$^+$ cells for 9247 and 5125.

(D) Flow cytometry plots show TRBV staining with one of 2 groups of 12 antibodies for individual 9247. Percentages of TRBV$^+$ cells are indicated. The sorted population is indicated. The positive group was further subdivided in (E).

(E) Flow cytometry plots show TRBV staining with one of 4 groups of 3 TRBV antibodies from individual 9247. Percentages indicate the fraction of TRBV$^+$ cells in each group. The sorted population is indicated. The clone of interest was only found in cells stained with the TRBV^H cocktail indicated in red and was further subdivided in (F).

(F) Flow cytometry plots show TRBV staining with 3 different monoclonal antibodies from individual 9247. Percentage indicates the fraction of TRBV4-3$^+$ cells.

(G) Flow cytometry plots show TRBV staining with antibody cocktails A–H from individual 5125. The clone of interest was only found in the TRBV^G cocktail, which was further subdivided in (H). Percentage indicates the fraction of TRBV$^+$ cells for each cocktail.

(H) Flow cytometry plots show TRBV staining with the 3 different antibodies in the TRBV^G cocktail from individual 5125. The clone of interest was found in the TRBV2$^+$ population. Percentage indicates the fraction of TRBV2$^+$ cells.

57% of all cells expressing the TCR associated with the expanded latent clone in the 6 individuals were found in cluster 7. Except for individual 9247, the fraction of CD4$^+$ T cells belonging to the latent clone in cluster 7 was greater than all other clusters ranging from 48% to 73% (Figure 5B; Table S6). In individual 9247, the largest fraction of latent cells was in cluster 6 (35%), which is closely related to cluster 7, and the second largest fraction of latent cells was in cluster 7 (29%).

CellPress
OPEN ACCESS

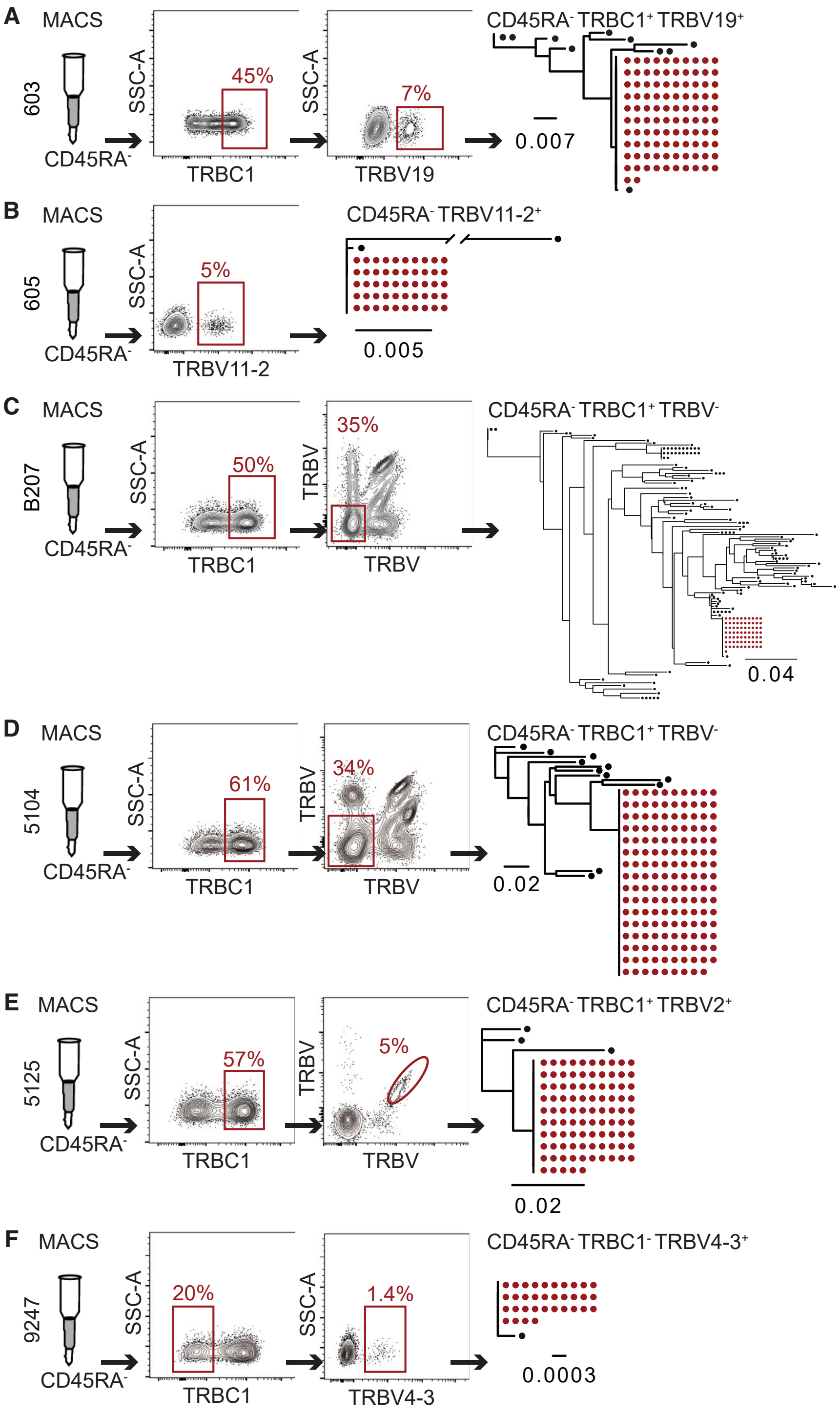

Figure 3. Combined enrichment based on CD45RA, TRBC, and TRBV expression in individuals 603, 605, B207, 5104, 5125, and 9247

Red outlines indicate the population containing the clone of interest. Maximum-likelihood phylogenetic trees of *env* sequences derived from sorted population, as indicated. *Env* gene of the clone of interest marked in red. The scale bars indicate the number of substitutions per site. Each sort was performed once for limiting dilution Env PCR amplification and sequencing and confirmed the previous screening for the respective marker of the latent clone of interest. See also Tables S3 and S4.

(A) Enrichment in individual 603 after purification of CD45RA$^-$ memory cells followed by staining and sorting for TRBC1$^+$ and TRBV19$^+$ cells. Flow cytometry plots show TRBC1 and TRBV19 staining. Percentages of CD45RA$^-$TRBC1$^+$ and CD45RA$^-$TRBC1$^+$TRBV19$^+$ cells are indicated.

(B) Enrichment in individual 605 after purification CD45RA$^-$ memory cells followed by staining TRBV11-2$^+$ cells, which were >90% TRBC1$^+$ (Figure S3B). Flow cytometry plot shows TRBV11-2 staining. Percentage of CD45RA$^-$TRBV11-2$^+$ cells is indicated.

(C) Enrichment in individual B207 after purification of CD45RA$^-$ memory cells followed by staining and sorting for TRBC1$^+$ and TRBV$^-$ cells. Flow cytometry plots show TRBC1 and combined 24 TRBV staining. Percentages of CD45RA$^-$TRBC1$^+$ and CD45RA$^-$TRBC1$^+$TRBV$^-$ cells are indicated.

(D) Enrichment in individual 5104 after purification CD45RA$^-$ memory cells followed by staining and sorting for TRBC1$^+$ and TRBV$^-$ cells. Flow cytometry plots show TRBC1 and TRBV staining. Percentages of CD45RA$^-$TRBC1$^+$ and CD45RA$^-$TRBC1$^+$TRBV$^-$ cells are indicated.

(E) Enrichment in individual 5125 after purification CD45RA$^-$ memory cells followed by staining and sorting for TRBC1$^+$ and TRBV2$^+$ cells. Flow cytometry plots show TRBC1 and TRBV2 staining. Percentages of CD45RA$^-$TRBC1$^+$ and CD45RA$^-$TRBC1$^+$TRBV2$^+$ cells are indicated.

(F) Enrichment in individual 9247 after purification of CD45RA$^-$ memory cells followed by staining and sorting for TRBC1- and TRBV4-3$^+$ cells. Flow cytometry plots show TRBC1 and TRBV4-3 staining. Percentages of CD45RA$^-$TRBC1$^-$ and CD45RA$^-$TRBC1$^-$TRBV4-3$^+$ cells are indicated.

Cluster 7 was enriched in clonally expanded cells which made up 58%–89% of all cells in the cluster (Table S7). To determine whether residence in cluster 7 is a general property of expanded clones of memory CD4$^+$ T cells, we determined the position of the cells in the next largest and/or smallest clones of CD4$^+$ memory T cells in the UMAP (Figure 5A). None of the 11 neighboring CD4$^+$ T cell clones examined were predominantly found in gene expression cluster 7. In addition, some TCR clones clustered by gene expression, whereas others did not. For example, in individual B207, cells in the next largest clone to the one containing latent proviruses were found in 8 of the 15 clusters. In contrast, cells in the next smaller clone in B207 were found primarily in gene expression cluster 10 (63%), which contains cells expressing *Foxp3* (Figure 5B; Table S6). We conclude that clonal expansion per se is not sufficient for CD4$^+$ memory T cell accumulation in gene expression cluster 7.

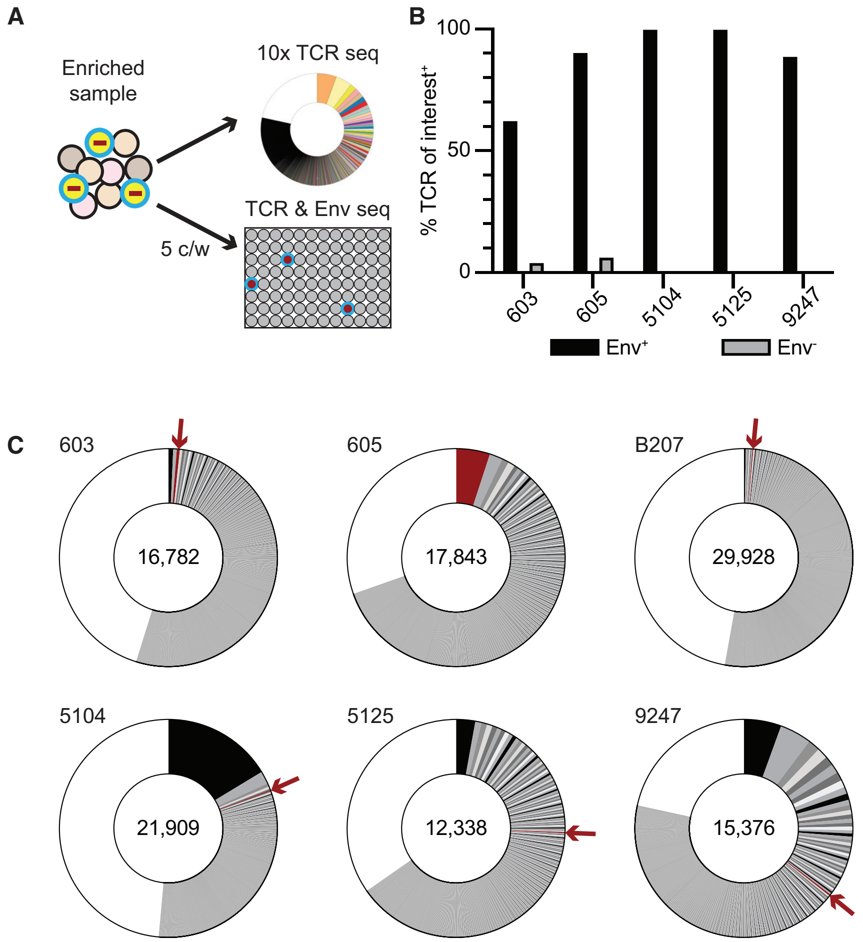

Figure 4. Identification of latent clone TCR sequences

(A) Limiting dilution sorting strategy to identify the TCR expressed by the CD4$^+$ T cells harboring the clone of interest. Each sample was enriched based on CD45RA, TRBC, TRBV expression, and 5 cells sorted per well (c/w) into microwell plates. *Env* sequencing identified wells that contained a cell of the latent clone of interest. TCRs were amplified and sequenced from all Env$^+$ and a selection of Env$^-$ wells in technical duplicates. Each sort was performed once.

(B) Bar graph shows the relevant TCR clonotypes identified in Env$^+$ and Env$^-$ wells. 603: Env$^+$ n = 8 and Env$^-$ n = 77; 605: Env$^+$ n = 21 and Env$^-$ n = 62; 5104: Env$^+$ n = 7 and Env$^-$ n = 36; 5125: Env$^+$ n = 4 and Env$^-$ n = 31; 9247: Env$^+$ n = 9 and Env$^-$ n = 42. See also Table S5.

(C) Pie charts show the relative size of TCR clones as slices. The areas indicated in white represent unique TCR sequences. The number on the left above the pie chart is the donor ID for each individual. The number in the center of the pie chart represents the number of cells assayed for each individual. The clone of interest is indicated by a red arrow and pie slice. See also Table S4.

To evaluate proviral transcription, HIV reads in the 10x gene-expression dataset were analyzed. HIV reads were detected in 58 cells that were found in all individuals except 5125. Six of those cells belonged to the latent clone of interest in individual 605. The remaining cells with HIV reads were not associated with any of the latent clones of interest. The number of HIV reads per cell ranged from 1 to 34 (mean 4.9). Those reads were, based on the unique molecular identifiers (UMIs), derived from 1 to 4 HIV transcripts per cell (mean 1.2). The 10x Genomics platform preferentially captures highly expressed genes, and proviral transcription is only found at a relatively low level in resting latent cells (Wiegand et al., 2017). Thus, HIV-expressing cells were difficult to identify reliably, and a comparison of HIV-expressing and transcriptionally silent latent cells was not possible.

Examination of the top 120 genes that define the UMAP clusters revealed that clusters 7 and 8 are closely related (Figure 6A). Cluster 7 is enriched in genes that encode antigen-presenting molecules or their chaperones such as *HLA-DR* and *HLA-DP*, as well as *CD74*, the invariant chain for major histocompatibility complex class II (MHC class II) molecules (Stumptner-Cuvelette and Benaroch, 2002) (Figure 6A; Table S8). In addition, cluster 7 is distinguished by expression of *CCL5*, granzymes A and K (*GZMA*, *GZMK*), cystatin F (*CST7*), and the nuclear proteins *LYAR* and *DUSP2*. Differential expression of some of these genes has been reported in activated latent CD4$^+$ T cells (Cohn et al., 2018; Horsburgh et al., 2020; Lee et al., 2019). However, granzyme A and K proteins do not accumulate specifically in latent cells because flow cytometry-based cell sorting for these markers did not enrich latent cells.

Cluster 7 shares many upregulated genes with cluster 8, namely *CCL5*, *CST7*, and *GZMA* (Figure 6A). The two closely related clusters differ in that cluster 8 cells also express *GZMB*, *GZMH*, perforin 1 (*PRF1*), natural killer cell granule protein 7 (*NKG7*), and granulysin (*GNLY*), each of which is associated with cytotoxic CD4$^+$ T cells (Hashimoto et al., 2019; Turman et al., 1993). This population of cells is frequently expanded in infection and chronic inflammation (Juno et al., 2017) and is also found enriched among tumor-infiltrating lymphocytes (Oh and Fong, 2021). Moreover, the relative proportion of cytotoxic CD4$^+$ T cells among memory cells is expanded in HIV-1-infected individuals, including those on suppressive antiretroviral therapy (ART) (Appay et al., 2002; Zaunders et al., 2004).

To examine the relationship between the cells in UMAP clusters and genes associated with CD4$^+$ T cell identity, we performed unsupervised clustering analysis with a collection of genes that are up- or downregulated in cytotoxic CD4$^+$ T cells (Appay et al., 2002; Cachot et al., 2021; Hashimoto et al., 2019; Juno et al., 2017; Takeuchi and Saito, 2017; Zaunders et al., 2004) (Figure 6B). In agreement with the UMAP, cluster 8 stands out as most closely related to CD4$^+$ cytotoxic cells, and cluster 7 is its closest relative. Nonetheless, cluster 7 differs from cytotoxic CD4$^+$ T cells in cluster 8 in several respects, including expression of higher levels of

CellPress
OPEN ACCESS

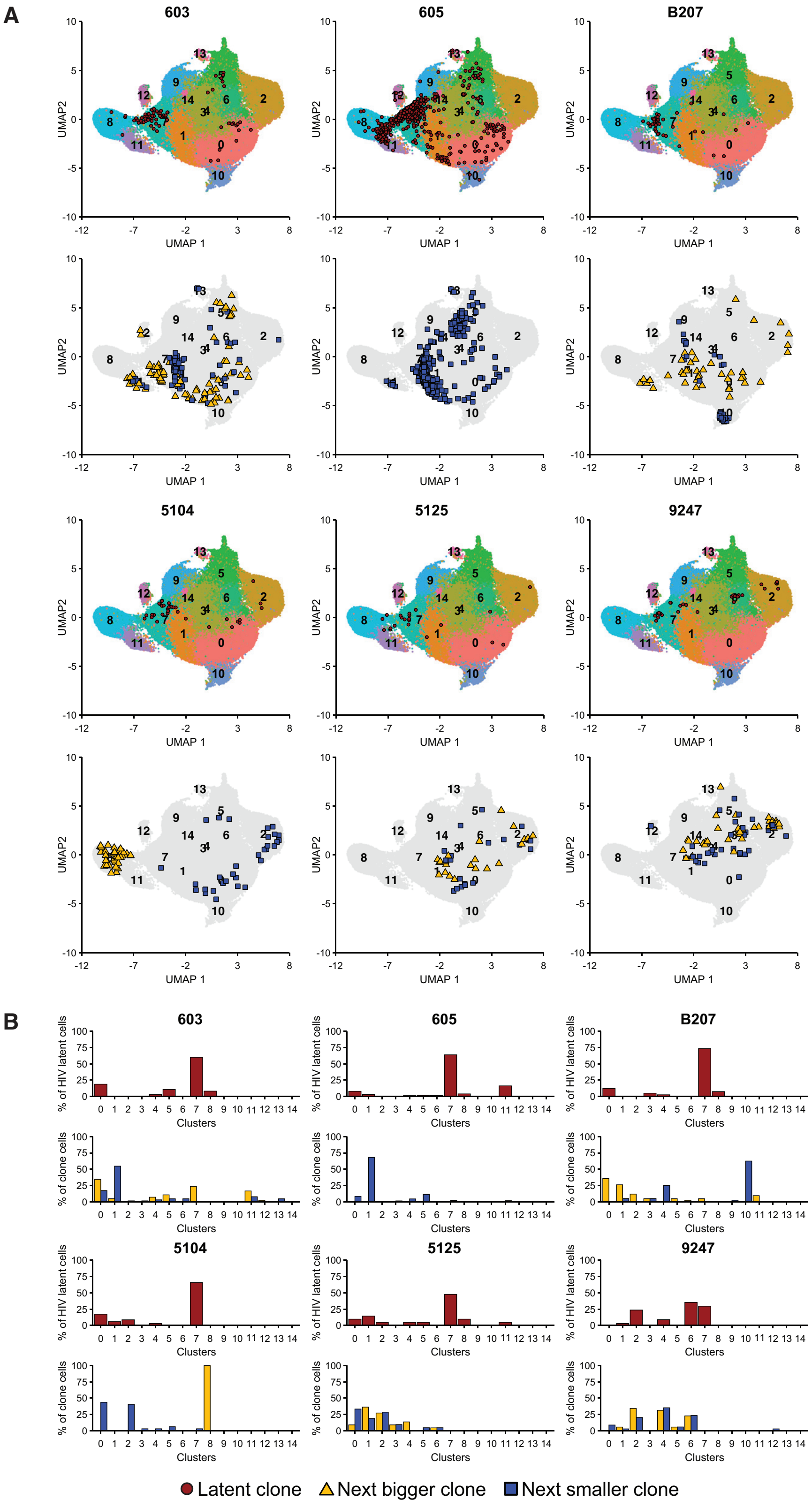

Figure 5. Uniform manifold approximation and projection (UMAP) of 10x gene-expression data

Data representing mRNA expression by 109,217 individual cells is shown. The latent clone of interest as well as the next biggest and next smallest clone in size were located in the UMAP by their TCR sequence. Enrichment and 10x Genomics gene expression and TCR sequencing were performed once for individuals 603, 605, and 5125 and twice for individuals B207, 5104, and 9247. See also Tables S6 and S7.

(A) UMAPs show the position of the cells expressing the latent clone TCR for each of the 6 individuals as red dots. Underneath, UMAPs show the position of the next biggest (yellow triangles) and the next smallest (blue squares) clones in size to the clone of interest. For individual 605, the latent clone of interest was the biggest clone, and only the next smallest clone is shown.

(B) The bar graphs show the fraction of cells in the latent clone (red bars) in each of the 15 UMAP clusters, and the fraction of cells of the next biggest (yellow bars) and the next smallest (blue bars) clones in size to the clone of interest in each of the 15 UMAP clusters (Table S6).

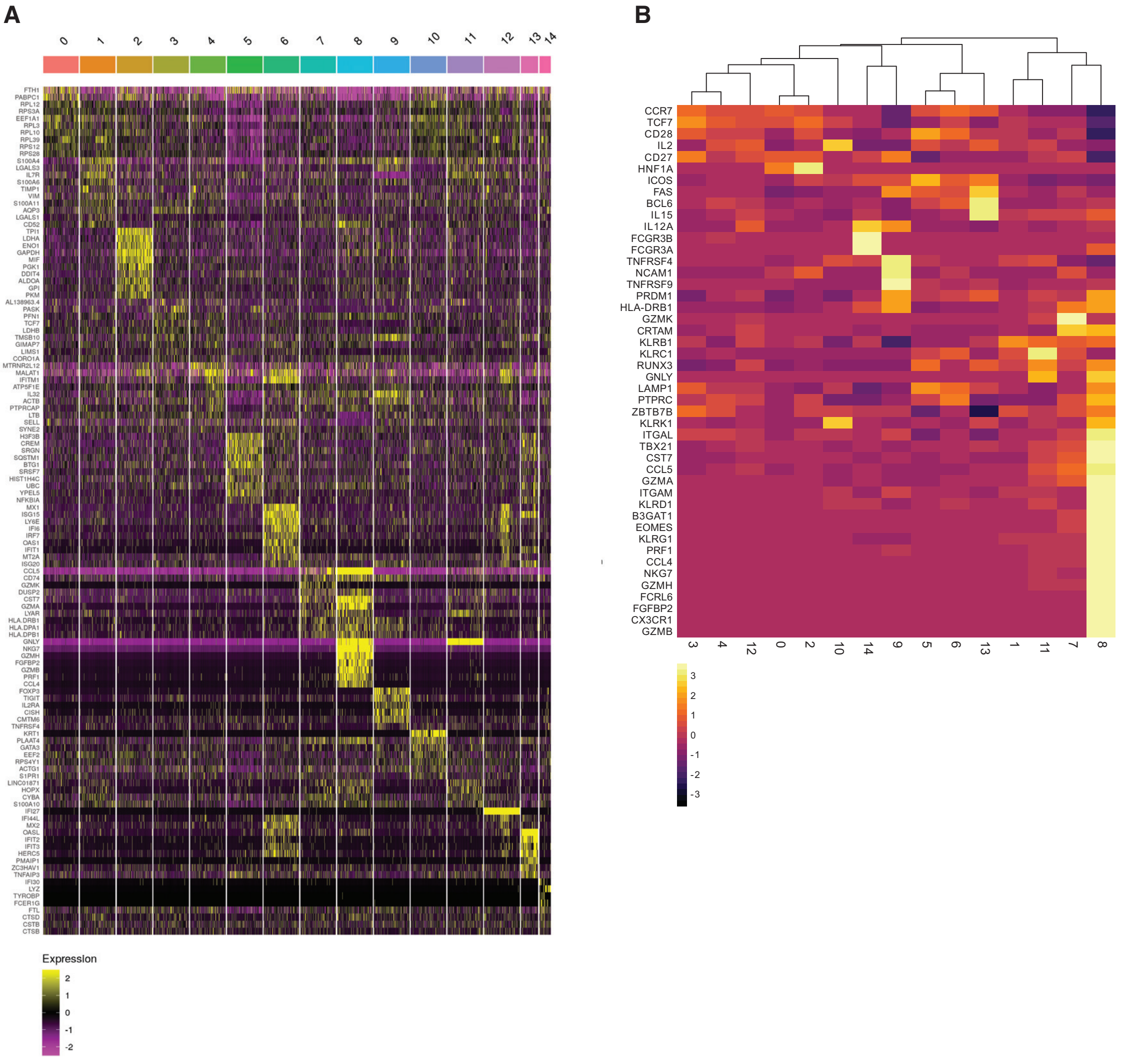

Figure 6. Heatmap of cluster-defining, differentially expressed genes

(A) Heatmap shows up to 10 of the most upregulated genes per UMAP cluster compared with all other clusters. Genes are indicated on the left and clusters above. Yellow shows relatively highly expressed genes and purple relatively downregulated genes. See also Table S8.

(B) Heatmap shows unsupervised clustering based on the expression of differentially expressed genes in cytotoxic CD4$^+$ T cells (Appay et al., 2002; Cachot et al., 2021; Hashimoto et al., 2019; Juno et al., 2017; Takeuchi and Saito, 2017; Zaunders et al., 2004). Genes are indicated on the left and clusters below. Yellow shows relatively highly expressed genes, and black shows relatively downregulated genes.

CD27, *CD28*, and *CCR7* (Appay et al., 2002) and lower levels of the transcription factors eomesodermin (*EOMES*) and *RUNX3* (Cachot et al., 2021; Juno et al., 2017; Takeuchi and Saito, 2017).

To further characterize the expanded clones of latent cells harboring intact HIV-1 proviruses, the 10x gene-expression data were projected on a reference dataset of a multimodal single-cell analysis of peripheral blood mononuclear cells (PBMCs) from HIV-1-negative individuals (Hao et al., 2021). Cluster 8 falls into the cytotoxic CD4$^+$ T cell (CTL) population, and cluster 7 falls into the CD4$^+$ T effector memory (T_{EM}) population that is marked by the expression of granzymes A and K, *DUSP2*, *CST7*, *LYAR*, and *HLA-DRB1* (Figure 7) (Hao et al., 2021). In four individuals, the overall fraction of cells in the clones harboring intact HIV-1 proviruses was greatest in the T_{EM} compartment (Figure 7). In two other individuals, 5104 and 9247, the clones were enriched in the central memory (T_{CM}) compartment. However, the T_{CM} population is larger than the T_{EM} compartment, and when corrected for compartment size, all individuals but individual 605 showed relative enrichment of the latent clone among T_{EM} cells (Table S9).

In conclusion, gene expression cluster 7 is characteristic of CD4$^+$ T_{EM} cells. This cluster harbors some of the features of CTLs but differs from cytotoxic cells in the expression in key transcriptional factors and components of the cytotoxic machinery (Juno et al., 2017; Takeuchi and Saito, 2017).

DISCUSSION

$CD4^+$ T cells harboring intact latent HIV-1 proviruses represent only a tiny fraction of all $CD4^+$ T cells. They are usually found among subsets of memory cells (Chomont et al., 2009; Hiener et al., 2017; Morcilla et al., 2021), but there are no specific markers that facilitate the purification of these cells (Cohn et al., 2020; Darcis et al., 2019). Consequently, their transcriptional program has only been studied after activation of HIV-1 expression, which thereby allows their identification (Baxter et al., 2016; Cohn et al., 2018; Liu et al., 2020; Neidleman et al., 2020; Pardons et al., 2019). We have devised a method to enrich quiescent latent memory $CD4^+$ T cells by means of their specific antigen receptors. We find that expanded clones of memory $CD4^+$ T cells that carry intact integrated HIV-1 proviruses are enriched among cells that express a transcriptional program that is found in $CD4^+$ T_{EM} cells. Our data are in accordance with studies showing enrichment of genetically intact proviruses in the $CD4^+$ T_{EM} compartment but extends previous observations by revealing the transcriptional program of resting latent cells (Duette et al., 2022; Hiener et al., 2017).

T_{CM} cells are antigen-experienced T cells that are $CD45RA^-$ $CD45RO^+CD27^+CCR7^+CD62L^+$ and circulate through secondary lymphoid organs (Chomont et al., 2009; Chtanova et al., 2005; Riou et al., 2007; Sallusto et al., 2004; Schiott et al., 2004; Weng et al., 2012). Upon re-stimulation with cognate antigen, T_{CM} cells secrete interleukin-2 (IL-2) and can differentiate further into T_{EM} cells that are polarized to secrete specific effector cytokines (Sallusto et al., 2004). T_{EM} cells are $CD45RA^-CD45RO^+CD27^-CCR7^-$ and express chemokine receptors that enable them to home to inflamed tissues (Chomont et al., 2009; Sallusto et al., 2004; Weng et al., 2012). These cells are more committed to specific T helper (T_H) differentiation lineages than T_{CM} cells and secrete effector cytokines or function as CTLs upon cognate antigen challenge (Farber et al., 2014; Riou et al., 2007; Sallusto et al., 2004). The observation that expanded clones of latent cells are frequently found in the $CD4^+$ T_{EM} compartment is consistent with the finding that clones of latent cells and T_{EM} cells develop in response to chronic viral infection (Farber et al., 2014; Harari et al., 2004, 2005; Mendoza et al., 2020; Simonetti et al., 2021; Stubbe et al., 2006; Weng et al., 2012).

$CD4^+$ cytotoxic T cells are a subset of T_{EM} cells whose effector function is killing target cells. Like other T_{EM} cells, they develop in response to chronic antigenic stimulation. They are prominently expanded in HIV-1, cytomegalovirus (CMV), Epstein-Barr virus (EBV) infection, chronic inflammatory diseases, and virally induced malignancies (Abana et al., 2017; Appay et al., 2002; Juno et al., 2017; Oh and Fong, 2021; Zaunders et al., 2004). Their polyfunctional phenotype is most closely associated with the Th1 phenotype, but they can also develop from other T cell lineages. The mechanism that regulates their development is not entirely defined but is associated with expression of *T-bet*, *EOMES*, *Runx3*, and *Blimp1* and downregulation of *ThPOK*, *Bcl6*, and *TCF1* (Cachot et al., 2021; Juno et al., 2017; Takeuchi and Saito, 2017). In keeping with the finding that clones of $CD4^+$ T cells harboring intact latent proviruses cluster in close proximity to $CD4^+$ cytotoxic cells, this subset of T_{EM} cells can respond to antigens found in chronic viral infections such as HIV-1, CMV, and EBV (Cameron et al., 2010; Gantner et al., 2020; Mendoza et al., 2020; Simonetti et al., 2021).

$CD4^+$ T cell-containing integrated proviruses in individuals on suppressive ART can express HIV-1 RNA, but the majority of these cells harbor defective proviruses (Bruner et al., 2016; Cho et al., 2022; Cohn et al., 2015; Einkauf et al., 2022; Eriksson et al., 2013; Hiener et al., 2017; Ho et al., 2013; Imamichi et al., 2016; Kearney et al., 2016; Lee et al., 2017; Peluso et al., 2020; Pollack et al., 2017). When examined based on HIV-1 RNA expression alone, irrespective of whether the provirus is intact, $CD4^+$ T cells containing integrated proviruses are enriched in granzyme B expression, suggestive of residence in the $CD4^+$ cytotoxic T cell compartment (Collora et al., 2022). Although we find a fraction of latent cells containing intact proviruses in the CTL compartment, this is a minority in 5 out of 6 individuals.

Members of a T cell clone expressing the same TCR can adopt different fates depending on several different factors including affinity, antigen concentration, and the cytokine milieu (Chang et al., 2007; Hale and Ahmed, 2015; Lee et al., 2017). Consistent with this idea, $CD4^+$ T cells expressing the TCR associated with latent proviruses are not entirely limited to a single gene-expression cluster. Nevertheless, the observation that a large fraction of the cells in the expanded clones we studied can be found in one specific transcriptional cluster among all memory $CD4^+$ T cells stands in contrast to other similarly sized expanded clones obtained from the same individuals. The latter are found in several different clusters that diverge between clones and individuals, and many of the cells in these clones are widely dispersed among clusters with different transcriptional signatures. For example, among the 11 random memory $CD4^+$ T cell clones of similar size examined in the 6 individuals, only 1 in individual 603 showed enrichment above 5% in cluster 7.

In chronically infected individuals, at least 50% of the cells carrying intact proviruses belong to expanded clones (Bui et al., 2017; Lorenzi et al., 2016), each of which can be distinguished by expression of a specific TCR that is associated with a unique proviral integration site (Cohn et al., 2015, 2018; Einkauf et al., 2019, 2022; Maldarelli et al., 2014; Simonetti et al., 2021; Wagner et al., 2014). The intact reservoir is dynamic, and while the absolute number of cells in the reservoir decreases slowly with a half-life of 4–5 years, clonality increases with time after infection in people on suppressive ART (Antar et al., 2020; Cho et al., 2022; Cohn et al., 2015; Crooks et al., 2015; Hosmane et al., 2017; Lee et al., 2017; Siliciano et al., 2003). However, clonal expansion is not a unique feature of $CD4^+$ T cells harboring intact proviruses. Defective proviruses are also found predominantly in expanded clones (Cho et al., 2022; Cohn et al., 2015; Imamichi et al., 2014), and clones of $CD4^+$ T cells are prominent among non-HIV-1-infected individuals (Qi et al., 2014).

Some of the genes that help define quiescent $CD4^+$ T cells in cluster 7 have also been reported to be expressed in latent cells, such as *HLA-DR* (Cohn et al., 2018; Horsburgh et al., 2020; Lee et al., 2017), or, like *CD2* or *LYAR*, upregulated in *in vitro* models of HIV infection and latency (DeBoer et al., 2014; Iglesias-Ussel et al., 2013). In contrast, *CCL5* is downregulated upon latent cell reactivation *in vitro* (Cohn et al., 2018; Iglesias-Ussel et al.,

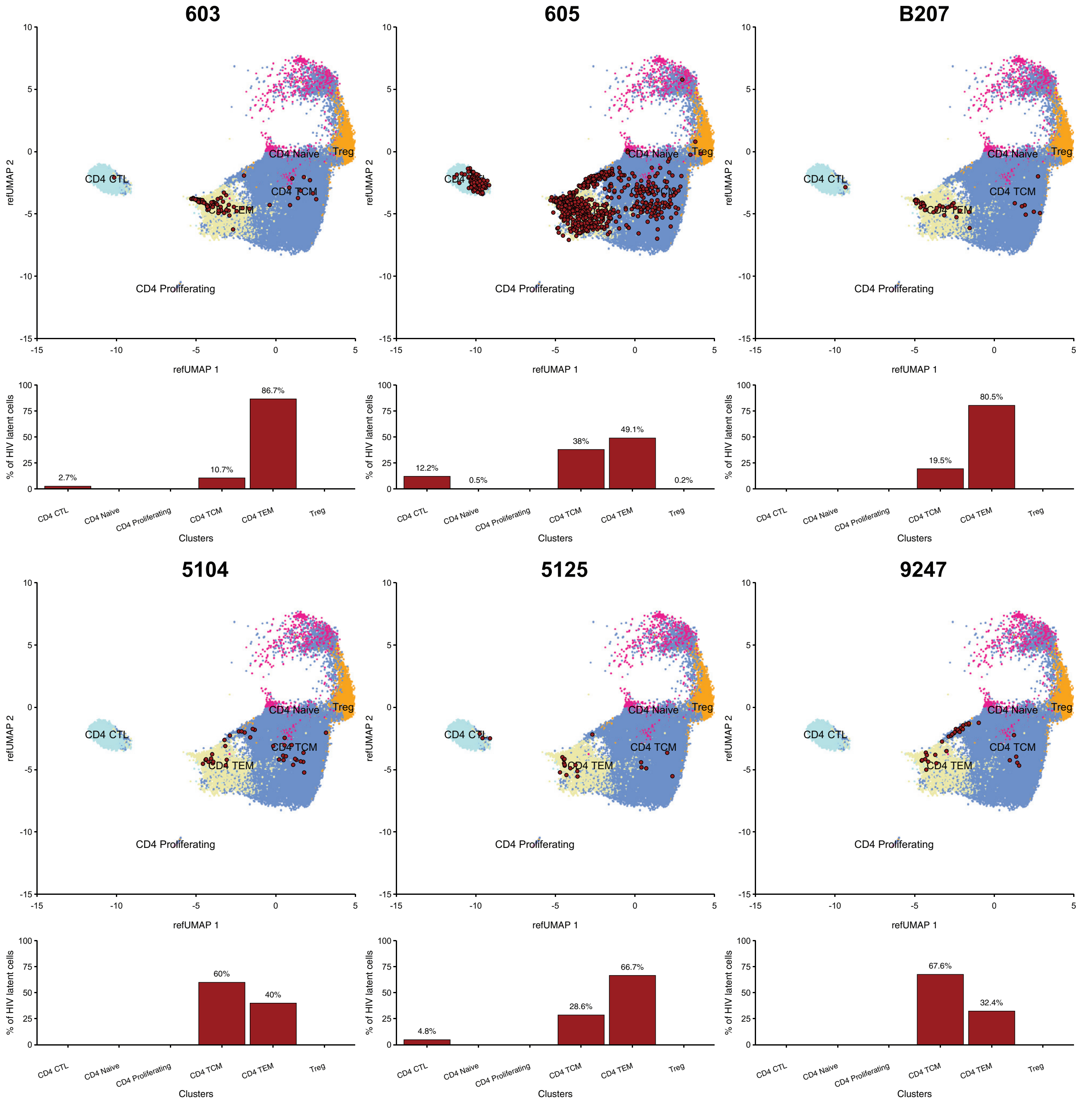

Figure 7. Projection of 10x gene-expression data on UMAP of CD4$^+$ T cells from multimodal single-cell sequencing

Projection of data representing mRNA expression by 109,217 individual cells on a multimodal UMAP of CD4$^+$ T cells from HIV-negative individuals (Hao et al., 2021). The latent clone of interest in each individual was identified by its TCR sequence and is represented as red dots. Underneath each UMAP, the bar graph shows the fraction of cells in the latent clone in each T cell subpopulation as indicated by the number above each bar. See also Table S9.

2013) but was upregulated in gene expression cluster 7. Notably, these genes had not been linked to a specific transcriptional program that differentiates latent cells from other CD4$^+$ T cells.

Cells expressing the genes that define cluster 7 are not a unique feature of HIV-1 infection and can be found in non-infected individuals (Hao et al., 2021; Hashimoto et al., 2019). Therefore, HIV-1 proviral integration and latency per se are not required for T cells to acquire the cluster 7 transcriptional program. Accordingly, clones in which all cells were latently infected (individuals 5104, 5125, and 9247) did not display different cluster patterns than clones in which only a fraction of cells was latently infected (individuals B207, 603, and 605). Thus, latent proviral integration does not induce a specific transcriptional profile.

Why latent proviruses are enriched in cells expressing this particular program is not known. One possibility among many is that the cluster 7 program favors suppression of HIV-1 gene expression during T cell activation, which would permit cell division in the absence of HIV-1 virion production and cell death. An alternative, but non-exclusive, possibility is that these cells are among the most likely to respond to a chronic infection and therefore the most likely to be infected and become latent and subsequently undergo clonal expansion in response to a persistent antigen. Cluster 7 shows increased expression of *CST7*, which is associated with reduced natural killer (NK) cytotoxicity and could help such cells evade elimination (Perisic Nanut et al., 2017).

Limitations of the study

Our analysis is limited to large, expanded clones of latent cells in 6 individuals and did not include non-circulating CD4$^+$ T cell subsets such as tissue-resident CD4$^+$ T cells (Sasson et al., 2020). Whether these observations also apply to less-expanded or tissue-resident populations of latent cells remains to be determined. Moreover, the depth of sequencing available on the 10x Genomics platform is also limiting, and therefore additional elements of the cluster 7 transcriptional program remain to be defined. Despite these caveats, the observation that latent cells preferentially display a specific transcriptional program suggests that these cells could be specifically targeted for elimination.

STAR★METHODS

Detailed methods are provided in the online version of this paper and include the following:

- KEY RESOURCES TABLE
- RESOURCE AVAILABILITY
  - Lead contact
  - Materials availability
  - Data and code availability
- EXPERIMENTAL MODEL AND SUBJECT DETAILS
  - Participant cohort
- METHOD DETAILS
  - Cell sorting
  - gDNA extraction and quantification
  - Near full-length proviral amplification and Q4PCR
  - Env PCR
  - Env2 PCR product and NFL2 PCR product sequencing
  - Phylogenetic trees
  - 10× genomics
  - Latent clone TCR identification
- QUANTIFICATION AND STATISTICAL ANALYSIS
  - Single-cell RNA-seq and single-cell TCR-seq processing
  - Mapping scRNA-seq to CD4$^+$ T cells reference
  - HIV-1 transcript detection using 10× data

SUPPLEMENTAL INFORMATION

Supplemental information can be found online at https://doi.org/10.1016/j.celrep.2022.111311.

ACKNOWLEDGMENTS

We thank all study participants who devoted time to our research, the Rockefeller University Hospital Research support office and nursing staff and all members of the M.C.N. laboratory for discussions, and M. Jankovic and G. Scrivanti for laboratory support. We thank C. Zhao, H. Duan, C. Lai, and S. Huang from the Genomics Resource Center at the Rockefeller University for preparing and sequencing the 10x Genomics libraries. We also thank K. Chhosphel and K.M. Gordon for operating the cell sorters. This work was supported by the National Institutes of Health (grants 1U01AI129825 to M.C. and UM1 AI100663 and R01AI129795 to M.C.N.); REACH Delaney (grant UM1 AI164565 to M.C.); the Einstein-Rockefeller-CUNY Center for AIDS Research (grant 1P30AI124414-01A1); BEAT-HIV Delaney (grant UM1 AI126620 to M.C.); and the Robertson Fund. L.B.C. is supported by the Delaney AIDS Research Enterprise (DARE) (1UM1AI126611-01), NIH REACH Martin Delaney Collaboratory (1UM1AI164565-01), and the Bill and Melinda Gates Foundation (INV-002707). C.G. was supported by the Robert S. Wennett postdoctoral fellowship, the Shapiro-Silverberg Fund for the Advancement of Translational Research, and by the National Center for Advancing Translational Sciences (National Institutes of Health Clinical and Translational Science Award program, grant UL1 TR001866). M.C.N. is a Howard Hughes Medical Institute (HHMI) Investigator. This article is subject to HHMI's Open Access to Publications policy. HHMI lab heads have previously granted a non-exclusive CC BY 4.0 license to the public and a sublicensable license to HHMI in their research articles. Pursuant to those licenses, the author-accepted manuscript of this article can be made freely available under a CC BY 4.0 license immediately upon publication.

AUTHOR CONTRIBUTIONS

G.H.J.W., Y.B.-O., L.B.C., M.J., and M.C.N. conceived and designed experiments. C.G. and M.C. recruited participants, supervised sample collection, and collected clinical data. G.H.J.W. performed the research. G.H.J.W., T.Y.O., V.R., L.B.C., M.J., and M.C.N analyzed data. T.Y.O. and V.R. performed bioinformatic analysis. H.H. and G.B. gave critical advice for cell sorting. G.H.J.W., L.B.C., M.J., and M.C.N. wrote the manuscript with help from all co-authors.

DECLARATION OF INTERESTS

Rockefeller University has patents on anti-HIV-1 antibodies 3BNC117 and 10-1074, on which M.C.N. is an inventor, that are licensed to Gilead.

Received: April 1, 2022
Revised: June 6, 2022
Accepted: August 12, 2022
Published: September 6, 2022

REFERENCES

Abana, C.O., Pilkinton, M.A., Gaudieri, S., Chopra, A., McDonnell, W.J., Wanjalla, C., Barnett, L., Gangula, R., Hager, C., Jung, D.K., et al. (2017). Cytomegalovirus (CMV) epitope-specific CD4(+) T cells are inflated in HIV(+) CMV(+) subjects. J. Immunol. *199*, 3187–3201.

Antar, A.A., Jenike, K.M., Jang, S., Rigau, D.N., Reeves, D.B., Hoh, R., Krone, M.R., Keruly, J.C., Moore, R.D., Schiffer, J.T., et al. (2020). Longitudinal study reveals HIV-1-infected CD4+ T cell dynamics during long-term antiretroviral therapy. J. Clin. Invest. *130*, 3543–3559.

Appay, V., Zaunders, J.J., Papagno, L., Sutton, J., Jaramillo, A., Waters, A., Easterbrook, P., Grey, P., Smith, D., McMichael, A.J., et al. (2002). Characterization of CD4(+) CTLs ex vivo. J. Immunol. *168*, 5954–5958.

Bachmann, N., von Siebenthal, C., Vongrad, V., Turk, T., Neumann, K., Beerenwinkel, N., Bogojeska, J., Fellay, J., Roth, V., Kok, Y.L., et al. (2019). Determinants of HIV-1 reservoir size and long-term dynamics during suppressive ART. Nat. Commun. *10*, 3193.

Baxter, A.E., Niessl, J., Fromentin, R., Richard, J., Porichis, F., Charlebois, R., Massanella, M., Brassard, N., Alsahafi, N., Delgado, G.G., et al. (2016). Single-cell characterization of viral translation-competent reservoirs in HIV-infected individuals. Cell Host Microbe *20*, 368–380.

Bruner, K.M., Murray, A.J., Pollack, R.A., Soliman, M.G., Laskey, S.B., Capoferri, A.A., Lai, J., Strain, M.C., Lada, S.M., Hoh, R., et al. (2016). Defective proviruses rapidly accumulate during acute HIV-1 infection. Nat. Med. *22*, 1043–1049.

Bruner, K.M., Wang, Z., Simonetti, F.R., Bender, A.M., Kwon, K.J., Sengupta, S., Fray, E.J., Beg, S.A., Antar, A.A.R., Jenike, K.M., et al. (2019). A quantitative approach for measuring the reservoir of latent HIV-1 proviruses. Nature *566*, 120–125.

Bruner, K.S., Robert (2018). Compositions and methods related to characterizing proviral reservoirs. United States Patent Application US15/568,893, filed April 22, 2016.

Bui, J.K., Sobolewski, M.D., Keele, B.F., Spindler, J., Musick, A., Wiegand, A., Luke, B.T., Shao, W., Hughes, S.H., Coffin, J.M., et al. (2017). Proviruses with identical sequences comprise a large fraction of the replication-competent HIV reservoir. PLoS Pathog. *13*, e1006283.

Cachot, A., Bilous, M., Liu, Y.C., Li, X., Saillard, M., Cenerenti, M., Rockinger, G.A., Wyss, T., Guillaume, P., Schmidt, J., et al. (2021). Tumor-specific cytolytic CD4 T cells mediate immunity against human cancer. Sci. Adv. *7*, eabe3348.

Cameron, P.U., Saleh, S., Sallmann, G., Solomon, A., Wightman, F., Evans, V.A., Boucher, G., Haddad, E.K., Sekaly, R.P., Harman, A.N., et al. (2010). Establishment of HIV-1 latency in resting CD4+ T cells depends on chemokine-induced changes in the actin cytoskeleton. Proc. Natl. Acad. Sci. USA *107*, 16934–16939.

Chang, J.T., Palanivel, V.R., Kinjyo, I., Schambach, F., Intlekofer, A.M., Banerjee, A., Longworth, S.A., Vinup, K.E., Mrass, P., Oliaro, J., et al. (2007). Asymmetric T lymphocyte division in the initiation of adaptive immune responses. Science *315*, 1687–1691.

Cho, A., Gaebler, C., Olveira, T., Ramos, V., Saad, M., Lorenzi, J.C.C., Gazumyan, A., Moir, S., Caskey, M., Chun, T.W., and Nussenzweig, M.C. (2022). Longitudinal clonal dynamics of HIV-1 latent reservoirs measured by combination quadruplex polymerase chain reaction and sequencing. Proc. Natl. Acad. Sci. USA *119*. e2117630119.

Chomont, N., El-Far, M., Ancuta, P., Trautmann, L., Procopio, F.A., Yassine-Diab, B., Boucher, G., Boulassel, M.R., Ghattas, G., Brenchley, J.M., et al. (2009). HIV reservoir size and persistence are driven by T cell survival and homeostatic proliferation. Nat. Med. *15*, 893–900.

Chtanova, T., Newton, R., Liu, S.M., Weininger, L., Young, T.R., Silva, D.G., Bertoni, F., Rinaldi, A., Chappaz, S., Sallusto, F., et al. (2005). Identification of T cell-restricted genes, and signatures for different T cell responses, using a comprehensive collection of microarray datasets. J. Immunol. *175*, 7837–7847.

Chun, T.W., Justement, J.S., Murray, D., Hallahan, C.W., Maenza, J., Collier, A.C., Sheth, P.M., Kaul, R., Ostrowski, M., Moir, S., et al. (2010). Rebound of plasma viremia following cessation of antiretroviral therapy despite profoundly low levels of HIV reservoir: implications for eradication. AIDS *24*, 2803–2808.

Chun, T.W., Stuyver, L., Mizell, S.B., Ehler, L.A., Mican, J.A., Baseler, M., Lloyd, A.L., Nowak, M.A., and Fauci, A.S. (1997). Presence of an inducible HIV-1 latent reservoir during highly active antiretroviral therapy. Proc. Natl. Acad. Sci. USA *94*, 13193–13197.

Cockerham, L.R., Siliciano, J.D., Sinclair, E., O'Doherty, U., Palmer, S., Yukl, S.A., Strain, M.C., Chomont, N., Hecht, F.M., Siliciano, R.F., et al. (2014). CD4+ and CD8+ T cell activation are associated with HIV DNA in resting CD4+ T cells. PLoS One *9*, e110731.

Cohn, L.B., Chomont, N., and Deeks, S.G. (2020). The biology of the HIV-1 latent reservoir and implications for cure strategies. Cell Host Microbe *27*, 519–530.

Cohn, L.B., da Silva, I.T., Valieris, R., Huang, A.S., Lorenzi, J.C.C., Cohen, Y.Z., Pai, J.A., Butler, A.L., Caskey, M., Jankovic, M., and Nussenzweig, M.C. (2018). Clonal CD4(+) T cells in the HIV-1 latent reservoir display a distinct gene profile upon reactivation. Nat. Med. *24*, 604–609.

Cohn, L.B., Silva, I.T., Oliveira, T.Y., Rosales, R.A., Parrish, E.H., Learn, G.H., Hahn, B.H., Czartoski, J.L., McElrath, M.J., Lehmann, C., et al. (2015). HIV-1 integration landscape during latent and active infection. Cell *160*, 420–432.

Collora, J.A., Liu, R., Pinto-Santini, D., Ravindra, N., Ganoza, C., Lama, J.R., Alfaro, R., Chiarella, J., Spudich, S., Mounzer, K., et al. (2022). Single-cell multiomics reveals persistence of HIV-1 in expanded cytotoxic T cell clones. Immunity *55*, 1013–1031.e7.

Crooks, A.M., Bateson, R., Cope, A.B., Dahl, N.P., Griggs, M.K., Kuruc, J.D., Gay, C.L., Eron, J.J., Margolis, D.M., Bosch, R.J., and Archin, N.M. (2015). Precise quantitation of the latent HIV-1 reservoir: implications for eradication strategies. J. Infect. Dis. *212*, 1361–1365.

Darcis, G., Berkhout, B., and Pasternak, A.O. (2019). The quest for cellular markers of HIV reservoirs: any color you like. Front. Immunol. *10*, 2251.

De Scheerder, M.A., Vrancken, B., Dellicour, S., Schlub, T., Lee, E., Shao, W., Rutsaert, S., Verhofstede, C., Kerre, T., Malfait, T., et al. (2019). HIV rebound is predominantly fueled by genetically identical viral expansions from diverse reservoirs. Cell Host Microbe *26*, 347–358.e7.

DeBoer, J., Jagadish, T., Haverland, N.A., Madson, C.J., Ciborowski, P., and Belshan, M. (2014). Alterations in the nuclear proteome of HIV-1 infected T-cells. Virology *468-470*, 409–420.

Douek, D.C., Brenchley, J.M., Betts, M.R., Ambrozak, D.R., Hill, B.J., Okamoto, Y., Casazza, J.P., Kuruppu, J., Kunstman, K., Wolinsky, S., et al. (2002). HIV preferentially infects HIV-specific CD4+ T cells. Nature *417*, 95–98.

Duette, G., Hiener, B., Morgan, H., Mazur, F.G., Mathivanan, V., Horsburgh, B.A., Fisher, K., Tong, O., Lee, E., Ahn, H., et al. (2022). The HIV-1 proviral landscape reveals Nef contributes to HIV-1 persistence in effector memory CD4+ T-cells. J. Clin. Invest. *132*, e154422.

Einkauf, K.B., Lee, G.Q., Gao, C., Sharaf, R., Sun, X., Hua, S., Chen, S.M., Jiang, C., Lian, X., Chowdhury, F.Z., et al. (2019). Intact HIV-1 proviruses accumulate at distinct chromosomal positions during prolonged antiretroviral therapy. J. Clin. Invest. *129*, 988–998.

Einkauf, K.B., Osborn, M.R., Gao, C., Sun, W., Sun, X., Lian, X., Parsons, E.M., Gladkov, G.T., Seiger, K.W., Blackmer, J.E., et al. (2022). Parallel analysis of transcription, integration, and sequence of single HIV-1 proviruses. Cell *185*, 266–282.e15.

Eriksson, S., Graf, E.H., Dahl, V., Strain, M.C., Yukl, S.A., Lysenko, E.S., Bosch, R.J., Lai, J., Chioma, S., Emad, F., et al. (2013). Comparative analysis of measures of viral reservoirs in HIV-1 eradication studies. PLoS Pathog. *9*, e1003174.

Farber, D.L., Yudanin, N.A., and Restifo, N.P. (2014). Human memory T cells: generation, compartmentalization and homeostasis. Nat. Rev. Immunol. *14*, 24–35.

Finzi, D., Blankson, J., Siliciano, J.D., Margolick, J.B., Chadwick, K., Pierson, T., Smith, K., Lisziewicz, J., Lori, F., Flexner, C., et al. (1999). Latent infection of CD4+ T cells provides a mechanism for lifelong persistence of HIV-1, even in patients on effective combination therapy. Nat. Med. *5*, 512–517.

Gaebler, C., Falcinelli, S.D., Stoffel, E., Read, J., Murtagh, R., Oliveira, T.Y., Ramos, V., Lorenzi, J.C.C., Kirchherr, J., James, K.S., et al. (2021). Sequence evaluation and comparative analysis of novel assays for intact proviral HIV-1 DNA. J. Virol. *95*, e01986-20.

Gaebler, C., Lorenzi, J.C.C., Oliveira, T.Y., Nogueira, L., Ramos, V., Lu, C.L., Pai, J.A., Mendoza, P., Jankovic, M., Caskey, M., and Nussenzweig, M.C. (2019). Combination of quadruplex qPCR and next-generation sequencing for qualitative and quantitative analysis of the HIV-1 latent reservoir. J. Exp. Med. *216*, 2253–2264.

Gaebler, C., Nogueira, L., Stoffel, E., Oliveira, T.Y., Breton, G., Millard, K.G., Turroja, M., Butler, A., Ramos, V., Seaman, M.S., et al. (2022). Prolonged viral suppression with anti-HIV-1 antibody therapy. Nature *606*, 368–374.

Gantner, P., Pagliuzza, A., Pardons, M., Ramgopal, M., Routy, J.P., Fromentin, R., and Chomont, N. (2020). Single-cell TCR sequencing reveals

CellPress
OPEN ACCESS

phenotypically diverse clonally expanded cells harboring inducible HIV proviruses during ART. Nat. Commun. *11*, 4089.

Grau-Expósito, J., Luque-Ballesteros, L., Navarro, J., Curran, A., Burgos, J., Ribera, E., Torrella, A., Planas, B., Badía, R., Martin-Castillo, M., et al. (2019). Latency reversal agents affect differently the latent reservoir present in distinct CD4+ T subpopulations. PLoS Pathog. *15*, e1007991.

Hale, J.S., and Ahmed, R. (2015). Memory T follicular helper CD4 T cells. Front. Immunol. *6*, 16.

Han, A., Glanville, J., Hansmann, L., and Davis, M.M. (2014). Linking T-cell receptor sequence to functional phenotype at the single-cell level. Nat. Biotechnol. *32*, 684–692.

Hao, Y., Hao, S., Andersen-Nissen, E., Mauck, W.M., 3rd, Zheng, S., Butler, A., Lee, M.J., Wilk, A.J., Darby, C., Zager, M., et al. (2021). Integrated analysis of multimodal single-cell data. Cell *184*, 3573–3587.e29.

Harari, A., Vallelian, F., and Pantaleo, G. (2004). Phenotypic heterogeneity of antigen-specific CD4 T cells under different conditions of antigen persistence and antigen load. Eur. J. Immunol. *34*, 3525–3533.

Harari, A., Vallelian, F., Meylan, P.R., and Pantaleo, G. (2005). Functional heterogeneity of memory CD4 T cell responses in different conditions of antigen exposure and persistence. J. Immunol. *174*, 1037–1045.

Hashimoto, K., Kouno, T., Ikawa, T., Hayatsu, N., Miyajima, Y., Yabukami, H., Terooatea, T., Sasaki, T., Suzuki, T., Valentine, M., et al. (2019). Single-cell transcriptomics reveals expansion of cytotoxic CD4 T cells in supercentenarians. Proc. Natl. Acad. Sci. USA *116*, 24242–24251.

Henrich, T.J., Hobbs, K.S., Hanhauser, E., Scully, E., Hogan, L.E., Robles, Y.P., Leadabrand, K.S., Marty, F.M., Palmer, C.D., Jost, S., et al. (2017). Human immunodeficiency virus type 1 persistence following systemic chemotherapy for malignancy. J. Infect. Dis. *216*, 254–262.

Hiener, B., Horsburgh, B.A., Eden, J.S., Barton, K., Schlub, T.E., Lee, E., von Stockenstrom, S., Odevall, L., Milush, J.M., Liegler, T., et al. (2017). Identification of genetically intact HIV-1 proviruses in specific CD4(+) T cells from effectively treated participants. Cell Rep. *21*, 813–822.

Ho, Y.C., Shan, L., Hosmane, N.N., Wang, J., Laskey, S.B., Rosenbloom, D.I.S., Lai, J., Blankson, J.N., Siliciano, J.D., and Siliciano, R.F. (2013). Replication-competent noninduced proviruses in the latent reservoir increase barrier to HIV-1 cure. Cell *155*, 540–551.

Horsburgh, B.A., Lee, E., Hiener, B., Eden, J.S., Schlub, T.E., von Stockenstrom, S., Odevall, L., Milush, J.M., Liegler, T., Sinclair, E., et al. (2020). High levels of genetically intact HIV in HLA-DR+ memory T cells indicates their value for reservoir studies. AIDS *34*, 659–668.

Hosmane, N.N., Kwon, K.J., Bruner, K.M., Capoferri, A.A., Beg, S., Rosenbloom, D.I.S., Keele, B.F., Ho, Y.C., Siliciano, J.D., and Siliciano, R.F. (2017). Proliferation of latently infected CD4(+) T cells carrying replication-competent HIV-1: potential role in latent reservoir dynamics. J. Exp. Med. *214*, 959–972.

Huang, A.S., Ramos, V., Oliveira, T.Y., Gaebler, C., Jankovic, M., Nussenzweig, M.C., and Cohn, L.B. (2021). Integration features of intact latent HIV-1 in CD4+ T cell clones contribute to viral persistence. J. Exp. Med. *218*, e20211427.

Iglesias-Ussel, M., Vandergeeten, C., Marchionni, L., Chomont, N., and Romerio, F. (2013). High levels of CD2 expression identify HIV-1 latently infected resting memory CD4+ T cells in virally suppressed subjects. J. Virol. *87*, 9148–9158.

Imamichi, H., Dewar, R.L., Adelsberger, J.W., Rehm, C.A., O'Doherty, U., Paxinos, E.E., Fauci, A.S., and Lane, H.C. (2016). Defective HIV-1 proviruses produce novel protein-coding RNA species in HIV-infected patients on combination antiretroviral therapy. Proc. Natl. Acad. Sci. USA *113*, 8783–8788.

Imamichi, H., Natarajan, V., Adelsberger, J.W., Rehm, C.A., Lempicki, R.A., Das, B., Hazen, A., Imamichi, T., and Lane, H.C. (2014). Lifespan of effector memory CD4+ T cells determined by replication-incompetent integrated HIV-1 provirus. AIDS *28*, 1091–1099.

Juno, J.A., van Bockel, D., Kent, S.J., Kelleher, A.D., Zaunders, J.J., and Munier, C.M.L. (2017). Cytotoxic CD4 T cells-friend or foe during viral infection? Front. Immunol. *8*, 19.

Kearney, M.F., Wiegand, A., Shao, W., Coffin, J.M., Mellors, J.W., Lederman, M., Gandhi, R.T., Keele, B.F., and Li, J.Z. (2016). Origin of rebound plasma HIV includes cells with identical proviruses that are transcriptionally active before stopping of antiretroviral therapy. J. Virol. *90*, 1369–1376.

Krishnan, V., and Zeichner, S.L. (2004). Host cell gene expression during human immunodeficiency virus type 1 latency and reactivation and effects of targeting genes that are differentially expressed in viral latency. J. Virol. *78*, 9458–9473.

Lassen, K.G., Hebbeler, A.M., Bhattacharyya, D., Lobritz, M.A., and Greene, W.C. (2012). A flexible model of HIV-1 latency permitting evaluation of many primary CD4 T-cell reservoirs. PLoS One *7*, e30176.

Lee, E., Bacchetti, P., Milush, J., Shao, W., Boritz, E., Douek, D., Fromentin, R., Liegler, T., Hoh, R., Deeks, S.G., et al. (2019). Memory CD4 + T-cells expressing HLA-DR contribute to HIV persistence during prolonged antiretroviral therapy. Front. Microbiol. *10*, 2214.

Lee, G.Q., Orlova-Fink, N., Einkauf, K., Chowdhury, F.Z., Sun, X., Harrington, S., Kuo, H.H., Hua, S., Chen, H.R., Ouyang, Z., et al. (2017). Clonal expansion of genome-intact HIV-1 in functionally polarized Th1 CD4+ T cells. J. Clin. Invest. *127*, 2689–2696.

Li, B., Gladden, A.D., Altfeld, M., Kaldor, J.M., Cooper, D.A., Kelleher, A.D., and Allen, T.M. (2007). Rapid reversion of sequence polymorphisms dominates early human immunodeficiency virus type 1 evolution. J. Virol. *81*, 193–201.

Liu, R., Yeh, Y.H.J., Varabyou, A., Collora, J.A., Sherrill-Mix, S., Talbot, C.C., Jr., Mehta, S., Albrecht, K., Hao, H., Zhang, H., et al. (2020). Single-cell transcriptional landscapes reveal HIV-1-driven aberrant host gene transcription as a potential therapeutic target. Sci. Transl. Med. *12*, eaaz0802.

Lorenzi, J.C.C., Cohen, Y.Z., Cohn, L.B., Kreider, E.F., Barton, J.P., Learn, G.H., Oliveira, T., Lavine, C.L., Horwitz, J.A., Settler, A., et al. (2016). Paired quantitative and qualitative assessment of the replication-competent HIV-1 reservoir and comparison with integrated proviral DNA. Proc. Natl. Acad. Sci. USA *113*, E7908–E7916.

Maldarelli, F., Wu, X., Su, L., Simonetti, F.R., Shao, W., Hill, S., Spindler, J., Ferris, A.L., Mellors, J.W., Kearney, M.F., et al. (2014). HIV latency. Specific HIV integration sites are linked to clonal expansion and persistence of infected cells. Science *345*, 179–183.

Mamedov, I.Z., Britanova, O.V., Zvyagin, I.V., Turchaninova, M.A., Bolotin, D.A., Putintseva, E.V., Lebedev, Y.B., and Chudakov, D.M. (2013). Preparing unbiased T-cell receptor and antibody cDNA libraries for the deep next generation sequencing profiling. Front. Immunol. *4*, 456.

Marini, A., Harper, J.M., and Romerio, F. (2008). An in vitro system to model the establishment and reactivation of HIV-1 latency. J. Immunol. *181*, 7713–7720.

Mendoza, P., Jackson, J.R., Oliveira, T.Y., Gaebler, C., Ramos, V., Caskey, M., Jankovic, M., Nussenzweig, M.C., and Cohn, L.B. (2020). Antigen-responsive CD4+ T cell clones contribute to the HIV-1 latent reservoir. J. Exp. Med. *217*, e20200051.

Morcilla, V., Bacchus-Souffan, C., Fisher, K., Horsburgh, B.A., Hiener, B., Wang, X.Q., Schlub, T.E., Fitch, M., Hoh, R., Hecht, F.M., et al. (2021). HIV-1 genomes are enriched in memory CD4(+) T-cells with short half-lives. mBio *12*, e0244721.

Neidleman, J., Luo, X., Frouard, J., Xie, G., Hsiao, F., Ma, T., Morcilla, V., Lee, A., Telwatte, S., Thomas, R., et al. (2020). Phenotypic analysis of the unstimulated in vivo HIV CD4 T cell reservoir. Elife *9*, e60933.

Nussenzweig, M.C., Shaw, A.C., Sinn, E., Danner, D.B., Holmes, K.L., Morse, H.C., 3rd, and Leder, P. (1987). Allelic exclusion in transgenic mice that express the membrane form of immunoglobulin mu. Science *236*, 816–819.

Oh, D.Y., and Fong, L. (2021). Cytotoxic CD4(+) T cells in cancer: expanding the immune effector toolbox. Immunity *54*, 2701–2711.

Palmer, S., Wiegand, A.P., Maldarelli, F., Bazmi, H., Mican, J.M., Polis, M., Dewar, R.L., Planta, A., Liu, S., Metcalf, J.A., et al. (2003). New real-time

reverse transcriptase-initiated PCR assay with single-copy sensitivity for human immunodeficiency virus type 1 RNA in plasma. J. Clin. Microbiol. *41*, 4531–4536.

Pardons, M., Baxter, A.E., Massanella, M., Pagliuzza, A., Fromentin, R., Dufour, C., Leyre, L., Routy, J.P., Kaufmann, D.E., and Chomont, N. (2019). Single-cell characterization and quantification of translation-competent viral reservoirs in treated and untreated HIV infection. PLoS Pathog. *15*, e1007619.

Peluso, M.J., Bacchetti, P., Ritter, K.D., Beg, S., Lai, J., Martin, J.N., Hunt, P.W., Henrich, T.J., Siliciano, J.D., Siliciano, R.F., et al. (2020). Differential decay of intact and defective proviral DNA in HIV-1-infected individuals on suppressive antiretroviral therapy. JCI Insight *5*, 132997.

Perišić Nanut, M., Sabotič, J., Švajger, U., Jewett, A., and Kos, J. (2017). Cystatin F affects natural killer cell cytotoxicity. Front. Immunol. *8*, 1459.

Pollack, R.A., Jones, R.B., Pertea, M., Bruner, K.M., Martin, A.R., Thomas, A.S., Capoferri, A.A., Beg, S.A., Huang, S.H., Karandish, S., et al. (2017). Defective HIV-1 proviruses are expressed and can Be recognized by cytotoxic T lymphocytes, which shape the proviral landscape. Cell Host Microbe *21*, 494–506.e4.

Qi, Q., Liu, Y., Cheng, Y., Glanville, J., Zhang, D., Lee, J.Y., Olshen, R.A., Weyand, C.M., Boyd, S.D., and Goronzy, J.J. (2014). Diversity and clonal selection in the human T-cell repertoire. Proc. Natl. Acad. Sci. USA *111*, 13139–13144.

Riou, C., Yassine-Diab, B., Van grevenynghe, J., Somogyi, R., Greller, L.D., Gagnon, D., Gimmig, S., Wilkinson, P., Shi, Y., Cameron, M.J., et al. (2007). Convergence of TCR and cytokine signaling leads to FOXO3a phosphorylation and drives the survival of CD4+ central memory T cells. J. Exp. Med. *204*, 79–91.

Salazar-Gonzalez, J.F., Bailes, E., Pham, K.T., Salazar, M.G., Guffey, M.B., Keele, B.F., Derdeyn, C.A., Farmer, P., Hunter, E., Allen, S., et al. (2008). Deciphering human immunodeficiency virus type 1 transmission and early envelope diversification by single-genome amplification and sequencing. J. Virol. *82*, 3952–3970.

Salazar-Gonzalez, J.F., Salazar, M.G., Keele, B.F., Learn, G.H., Giorgi, E.E., Li, H., Decker, J.M., Wang, S., Baalwa, J., Kraus, M.H., et al. (2009). Genetic identity, biological phenotype, and evolutionary pathways of transmitted/founder viruses in acute and early HIV-1 infection. J. Exp. Med. *206*, 1273–1289.

Sallusto, F., Geginat, J., and Lanzavecchia, A. (2004). Central memory and effector memory T cell subsets: function, generation, and maintenance. Annu. Rev. Immunol. *22*, 745–763.

Sasson, S.C., Gordon, C.L., Christo, S.N., Klenerman, P., and Mackay, L.K. (2020). Local heroes or villains: tissue-resident memory T cells in human health and disease. Cell. Mol. Immunol. *17*, 113–122.

Schiött, A., Lindstedt, M., Johansson-Lindbom, B., Roggen, E., and Borrebaeck, C.A.K. (2004). CD27- CD4+ memory T cells define a differentiated memory population at both the functional and transcriptional levels. Immunology *113*, 363–370.

Schmid, A., Gianella, S., von Wyl, V., Metzner, K.J., Scherrer, A.U., Niederöst, B., Althaus, C.F., Rieder, P., Grube, C., Joos, B., et al. (2010). Profound depletion of HIV-1 transcription in patients initiating antiretroviral therapy during acute infection. PLoS One *5*, e13310.

Sengupta, S., and Siliciano, R.F. (2018). Targeting the latent reservoir for HIV-1. Immunity *48*, 872–895.

Siliciano, J.D., Kajdas, J., Finzi, D., Quinn, T.C., Chadwick, K., Margolick, J.B., Kovacs, C., Gange, S.J., and Siliciano, R.F. (2003). Long-term follow-up studies confirm the stability of the latent reservoir for HIV-1 in resting CD4+ T cells. Nat. Med. *9*, 727–728.

Simonetti, F.R., Zhang, H., Soroosh, G.P., Duan, J., Rhodehouse, K., Hill, A.L., Beg, S.A., McCormick, K., Raymond, H.E., Nobles, C.L., et al. (2021). Antigen-driven clonal selection shapes the persistence of HIV-1-infected CD4+ T cells in vivo. J. Clin. Invest. *131*, 145254.

Song, L., Cohen, D., Ouyang, Z., Cao, Y., Hu, X., and Liu, X.S. (2021). TRUST4: immune repertoire reconstruction from bulk and single-cell RNA-seq data. Nat. Methods *18*, 627–630.

Stubbe, M., Vanderheyde, N., Goldman, M., and Marchant, A. (2006). Antigen-specific central memory CD4+ T lymphocytes produce multiple cytokines and proliferate in vivo in humans. J. Immunol. *177*, 8185–8190.

Stumptner-Cuvelette, P., and Benaroch, P. (2002). Multiple roles of the invariant chain in MHC class II function. Biochim. Biophys. Acta *1542*, 1–13.

Takeuchi, A., and Saito, T. (2017). CD4 CTL, a cytotoxic subset of CD4(+) T cells, their differentiation and function. Front. Immunol. *8*, 194.

Telwatte, S., Morón-López, S., Aran, D., Kim, P., Hsieh, C., Joshi, S., Montano, M., Greene, W.C., Butte, A.J., Wong, J.K., and Yukl, S.A. (2019). Heterogeneity in HIV and cellular transcription profiles in cell line models of latent and productive infection: implications for HIV latency. Retrovirology *16*, 32.

Turman, M.A., Yabe, T., McSherry, C., Bach, F.H., and Houchins, J.P. (1993). Characterization of a novel gene (NKG7) on human chromosome 19 that is expressed in natural killer cells and T cells. Hum. Immunol. *36*, 34–40.

Wagner, T.A., McLaughlin, S., Garg, K., Cheung, C.Y.K., Larsen, B.B., Styrchak, S., Huang, H.C., Edlefsen, P.T., Mullins, J.I., and Frenkel, L.M. (2014). HIV latency. Proliferation of cells with HIV integrated into cancer genes contributes to persistent infection. Science *345*, 570–573.

Weng, N.P., Araki, Y., and Subedi, K. (2012). The molecular basis of the memory T cell response: differential gene expression and its epigenetic regulation. Nat. Rev. Immunol. *12*, 306–315.

Wiegand, A., Spindler, J., Hong, F.F., Shao, W., Cyktor, J.C., Cillo, A.R., Halvas, E.K., Coffin, J.M., Mellors, J.W., and Kearney, M.F. (2017). Single-cell analysis of HIV-1 transcriptional activity reveals expression of proviruses in expanded clones during ART. Proc. Natl. Acad. Sci. USA *114*, E3659–E3668.

Wong, J.K., Hezareh, M., Günthard, H.F., Havlir, D.V., Ignacio, C.C., Spina, C.A., and Richman, D.D. (1997). Recovery of replication-competent HIV despite prolonged suppression of plasma viremia. Science *278*, 1291–1295.

Zaunders, J.J., Dyer, W.B., Wang, B., Munier, M.L., Miranda-Saksena, M., Newton, R., Moore, J., Mackay, C.R., Cooper, D.A., Saksena, N.K., and Kelleher, A.D. (2004). Identification of circulating antigen-specific CD4+ T lymphocytes with a CCR5+, cytotoxic phenotype in an HIV-1 long-term nonprogressor and in CMV infection. Blood *103*, 2238–2247.

CellPress
OPEN ACCESS

STAR★METHODS

KEY RESOURCES TABLE

REAGENT or RESOURCE	SOURCE	IDENTIFIER
Antibodies		
Anti-human CD3 PB	BioLegend	cat. 300431; RRID AB_1595437
Anti-human CD4 PerCP-Cy5.5	BioLegend	cat. 317428; RRID AB_1186122
Anti-human CD45RA PE-TR	ThermoFisher	cat. MHCD45RA17; RRID AB_10372222
Anti-human TCR Cβ1 BV605	BD	cat. 747979; RRID AB_2872440
Anti-human TCR Vβ17 FITC	Beckman Coulter	cat. IM1234; RRID AB_131007
Anti-human TCR Vβ21.3 FITC	Beckman Coulter	cat. IM1483; RRID AB_131021
Anti-human TCR Vβ7.2 FITC	Beckman Coulter	cat. B06666
Beta Mark TCR Vbeta Repertoire Kit	Beckman Coulter	cat. IM3497
Chemicals, peptides, and recombinant proteins		
Recombinant Proteinase K Solution	ThermoFisher	cat. AM2548
Phenol-chloroform-isoamyl alcohol mixture	Sigma-Aldrich	cat. 77617
GlycoBlue™ Coprecipitant	ThermoFisher	cat. AM9515
1× TE Buffer	ThermoFisher	cat. 12090015
Platinum™ Taq DNA Polymerase High Fidelity	Fisher Scientific	cat. 11-304-029
TaqMan Universal PCR Master Mix	ThermoFisher	cat. 4304437
Buffer TCL	Qiagen	cat. 1031576
2-Mercaptoethanol	Sigma-Aldrich	cat. 63689–25ML-F
Agencourt RNAClean XP	Beckman Coulter	cat. A63987
SuperScript™ III Reverse Transcriptase	ThermoFisher	cat. 18080044
RNasin® Plus Ribonuclease Inhibitor	Promega	cat. N2615
HotStarTaq DNA polymerase	Qiagen	cat. 203209
E-Gel™ 96 Agarose Gels with SYBR™ Safe DNA Gel Stain, 1%	ThermoFisher	cat. G720841
Illumina Tagment DNA TDE1 Enzyme and Buffer	Illumina	cat. 20034198
KAPA HiFi HotStart ReadyMix PCR Kit	Roche	cat. 07958935001
Critical commercial assays		
$CD4^+$ T cell isolation kit	Miltenyi	cat. 130-059-901
CD45RA MicroBeads	Miltenyi	cat. 130-045-901
MiSeq Reagent Kit v2 (300-cycles)	Illumina	cat. MS-102-2002
Miseq Reagent Kit v3 (600 cycle)	Illumina	cat. MS-102-3003
Qubit™ dsDNA BR Assay Kit	ThermoFisher	cat. Q32853
NucleoSpin Gel and PCR Clean-up kit	Quiagen	cat. 740609.50
Chromium Single Cell 5′ Library & Gel Bead Kit	10x Genomics	cat. PN-1000014
Chromium Single Cell V(D)J Enrichment Kit, Human T Cell	10x Genomics	cat. PN-1000005
NovaSeq 6000 S1 (100 cycles)	Illumina	cat. 20012865
NextSeq 500/550 Mid Output Kit v2.5 (300 Cycles)	Illumina	cat. 20024905
Deposited data		
Single cell RNA-Seq and TCR data	This paper	NCBI GEO: GSE204756
HIV-1 Envelope sequences	This paper	GenBank: ON662322 - ON664914

(Continued on next page)

Continued

REAGENT or RESOURCE	SOURCE	IDENTIFIER
Raw FASTQ TCR sequences	This paper	NCBI SRA: SRR19524296 - SRR19524298
**Oligonucleotides**		
See Table S10		
**Software and algorithms**		
Code for single-cell analysis	This paper	https://doi.org/10.5281/zenodo.6950427
Code to identify the TCR of the latent clone of interest	This paper	https://doi.org/10.5281/zenodo.6954076
FACSDiva software (version 2.0.2)	BD	https://www.bdbiosciences.com/en-us/products/software/instrument-software/bd-facsdiva-software
FlowJo (version 10.8.1)	BD	https://www.flowjo.com/solutions/flowjo/downloads
Defective and Intact HIV Genome Assembler	(Gaebler et al., 2019)	https://github.com/stratust/DIHIVA
QuantStudio Real-Time PCR Software version 1.3	ThermoFisher	https://www.thermofisher.com/us/en/home/global/forms/life-science/quantstudio-3-5-software.html
Geneious Prime software (version 11.0.12)	Biomatters	https://www.geneious.com/prime/
GraphPad Prism 9 (version 9.3.1)	GraphPad Software, LLC.	https://www.graphpad.com
**Other**		
Fixable Viability Dye eFluor 780	Invitrogen	cat. 65-0865-14
FcR Blocking Reagent, human	Miltenyi	cat. 130-059-901<br>RRID AB_2892112
Phase Lock Gel light	QuantaBio	cat. 10847–800
MiSeq System	Illumina	cat. SY-410-1003
FACS Aria IIu	BD	N/A
FACS Aria III	BD	N/A

RESOURCE AVAILABILITY

Lead contact

Further information and requests for resources and reagents should be directed to and will be fulfilled by the lead contact, Michel Nussenzweig (nussen@rockefeller.edu).

Materials availability

This study did not generate new unique reagents.

Data and code availability

- The data reported in this paper is publicly available as of the date of publication and archived at the following databases: Single cell RNA-Seq and TCR data is available at NCBI GEO: GSE204756; Envelope sequences deposited into the Genbank: ON662322 - ON664914; raw FASTQ sequences used to identify the TCR of latent HIV-1 cells are available at NCBI SRA: SRR19524296 - SRR19524298. Accession numbers are listed in the key resources table.
- All original code has been deposited publicly available as of the date of publication at the following repositories: The code for single-cell analysis was released at https://doi.org/10.5281/zenodo.6950427. The code to identify the TCR of the latent clone of interest is available on github (https://github.com/victor-ramos/demultiplex_and_assembly_TCR) and at https://doi.org/10.5281/zenodo.6954076. DOIs are listed in the key resources table.
- Any additional information required to reanalyze the data reported in this paper is available from the lead contact upon request.

EXPERIMENTAL MODEL AND SUBJECT DETAILS

Participant cohort

Study participants were recruited at the Rockefeller University hospital and gave informed written consent before participation in the study. The study protocols and procedures (MCA-0966, TSC-0910) met the standards of Good Clinical Practice and were approved by the institutional review board of the Rockefeller University. The participants' characteristics, including age and sex, are available in Table S1. An expanded intact latent clone was present in the latent reservoir of each individual (Figure S1, Table S2).

After leukapheresis, peripheral blood mononuclear cells (PBMCs) were isolated by Ficoll separation and stored in aliquots in liquid nitrogen. At the time of sample collection, all study participants were receiving ART, were aviremic and had not undergone experimental treatment regimes.

METHOD DETAILS

Cell sorting

All procedures were performed while maintaining cells at 4°C. $CD4^+$ T cells were negatively selected from PBMCs with the $CD4^+$ T cell isolation kit (Miltenyi, cat. 130-059-901). For memory cell separation, $CD45RA^-$ T cells were negatively selected using magnetic CD45RA MicroBeads (Miltenyi, cat. 130-045-901).

$CD4^+CD45RA^-$ T cells were incubated with Fc-blocking reagent for 10 min (Miltenyi, cat. 130-059-901). Fixable Viability Dye eFluor 780 (Invitrogen, cat. 65-0865-14) was used for live/dead cell staining (dilution 1:1,000). The following antibodies were used for surface staining (dilution 1:100): PerCP/Cy5.5 anti-human CD4 (BioLegend, cat. 317428), PacificBlue anti-human CD3 (BioLegend, cat. 300431), Brilliant Violet 605 anti-human TCR Cβ1 (BD, cat. 747979), FITC anti-human TCR Vβ17 (Beckman Coulter, cat. IM1234), FITC anti-human TCR Vβ21.3 (Backman Coulter, cat. IM1483), FITC anti-human TCR Vβ7.2 (Beckman Coulter, cat. B06666), and Beta Mark TCR Vbeta Repertoire Kit (Beckman Coulter, cat. IM3497). The Vbeta Repertoire Kit contains 24 different anti-TRBV antibodies that come in 8 vials (A – H). In each vial, there are three antibodies conjugated with either PE, or FITC, or PE-FITC.

For enrichment, the following population was sorted: individuals B207 and 5104 $CD3^+CD4^+TRBC1^+TRBV^-$ lymphocytes; individual 603 $CD4^+TRBC1^+TRBV19^+$ lymphocytes; individual 605 $CD4^+TRBV11\text{-}2^+$ lymphocytes; individual 5125 $CD4^+TRBC1^+TRBV2^+$ lymphocytes; individual 9247 $CD4^+TRBC1^-TRBV4\text{-}3^+$ lymphocytes. Sorts were performed on BD FACS Aria IIu and BD FACS Aria III using FACSDiva software (version 2.0.2, BD). Flowcytometry data was analyzed with FlowJo (version 10.8.1, BD)

gDNA extraction and quantification

Sorted cells were incubated with Proteinase K buffer (10 mM Tris, pH 8, 0.5% SDS, 100 mM NaCl, and 1 mM EDTA) and 250 μg/mL Proteinase K (ThermoFisher, cat. AM2548) at 50°C for 6 h. An equal volume of phenol-chloroform-isoamyl (Sigma-Aldrich, cat. 77617) was added and gDNA was extracted with Phase Lock Gel light tubes (QuantaBio, cat. 10847-800). gDNA was precipitated with ethanol and GlycoBlue Coprecipitant (ThermoFisher, cat. AM9515), washed twice with 70% ethanol and resuspended in TE buffer. gDNA concentration was measured by Qubit dsDNA BR Assay Kit (ThermoFisher, cat. Q32853).

Near full-length proviral amplification and Q4PCR

Near full-length proviral (NFL) amplification was performed as previously described (Gaebler et al., 2019).

The first round of near full-length proviral amplification (NFL1 PCR) was run in a 20 μL reaction volume with 0.5 U of Platinum Taq DNA Polymerase High Fidelity (Fisher Scientific, cat. 11-304-029) and outer PCR primers BLOuterF (5′-AAATCTCTAGCAG TGGCGCCCGAACAG-3′) and BLOuterR (5′-TGAGGGATCTCTAGTTACCAGAGTC-3′) (0.2 μM each) (Li et al., 2007), 2 mM $MgSO_4$ and 0.2 mM dNTPs at 94°C for 2 min; (94°C for 30 s, 64°C for 30 s, and 68°C for 10 min) for 3 cycles; (94°C for 30 s, 61°C for 30 s, and 68°C for 10 min) for 3 cycles; (94°C for 30 s, 58°C for 30 s, and 68°C for 10 min) for 3 cycles; (94°C for 30 s, 55°C for 30 s, and 68°C for 10 min) for 41 cycles; and then 68°C for 10 min as a touch down PCR.

Next, a quadruplex qPCR in a total reaction volume of 10 μL was performed. To 5 μL of TaqMan Universal PCR Master Mix, (ThermoFisher, cat. 4304437), 1 μL of the NFL1 PCR product and 4 μL of a master mix containing the following primer pairs and internal probes were added (final concentration in 10 μL reaction volume in brackets): PS F (5′-TCTCTCGACGCAGGACTC-3′) and PS R (5′-TCTAGCCTCCGCTAGTCAAA-3′) (675 nM each), PS probe (5′/Cy5/TTTGGCGTA/TAO/CTCACCAGTCGCC/3′/IAbRQSp (187.5 nM) (Bruner and Robert, 2018); env F (5′-AGTGGTGCAGAGAGAAAAAAGAGC-3′) and env R (5′-GTCTGGCCTGTACCGT CAGC-3′) (90 nM each), env probe (5′/VIC/CCTTGGGTTCTTGGGA/3′/MGB) (25 nM) (Bruner et al., 2019); gag F (5′-ATGTTTTCAG CATTATCAGAAGGA-3′) and gag R (5′-TGCTTGATGTCCCCCCACT-3′) (337.5 nM each), gag probe (5′/6-FAM/CCACCCC AC/ZEN/AAGATTTAAACACCATGCTAA/3′/IABkFQ) (93.75 nM) (Palmer et al., 2003); pol F (5′-GCACTTTAAATTTTCCCATTA GTCCTA-3′) and pol R (5′-CAAATTTCTACTAATGCTTTTATTTTTTC-3′) (675 nM each), pol probe (5′/NED/AAGCCAGGAATGGA TGGCC/3′/MGB) (187.5 nM) (Schmid et al., 2010). qPCR was performed using the Applied Biosystem QuantStudio 6 Flex Real-Time PCR System with the following conditions:

94°C for 10 min; (94°C for 15 s, 60°C for 1 min) for 40 cycles.

QuantStudio Real-Time PCR Software version 1.3 (ThermoFisher) was used for qPCR data analysis. Baseline correction (start cycle 3, end cycle 10) and normalized reported signal threshold (ΔRn threshold = 0.025) was set for all targets/probes. A fluorescent signal above the threshold between cycle value 10 and 40 of any two or more probes was identified and the corresponding NFL1 PCR samples were selected for NFL2 PCR.

NFL2 PCR was run in a 20 μL reaction volume with 1 μL of NFL1 PCR product, 0.5 U of Platinum Taq DNA Polymerase High Fidelity (Fisher Scientific, cat. 11-304-029) and inner PCR primers 275F (5′-ACAGGGACCTGAAAGCGAAAG-3′) and 280R (5′-CTAGTTAC CAGAGTCACACAACAGACG-3) (0.2 μM each) (Ho et al., 2013), 2 mM $MgSO_4$ and 0.2 mM dNTPs at 94°C for 2 min; (94 °C for 30 s, 64 °C for 30 s, and 68 °C for 10 min) for 3 cycles; (94 °C for 30 s, 61 °C for 30 s, and 68 °C for 10 min) for 3 cycles; (94°C for

30 s, 58°C for 30 s, and 68°C for 10 min) for 3 cycles; (94°C for 30 s, 55°C for 30 s, and 68°C for 10 min) for 41 cycles; and then 68°C for 10 min as a touch down PCR. NFL2 PCR products were then sequenced.

For each sample, a serial dilution of gDNA was performed to achieve a fluorescent signal above the threshold between cycle value 10 and 40 of any two or more probes in <30% of wells.

Env PCR

Envelope gene amplification was performed as previously described (Salazar-Gonzalez et al., 2008, 2009).

The first round of *env* amplification (Env1 PCR) for individuals 603, B207, 5104, 5125, and 9247 was run in a 20 μL reaction volume with 0.5 U of Platinum Taq DNA Polymerase High Fidelity (Fisher Scientific, cat. 11-304-029) and outer PCR primers Env5out (5′-TA GAGCCCTGGAAGCATCCAGGAAG-3′) and Env3out (5′-TTGCTACTTGTGATTGCTCCATGT-3′) (0.2 μM each) (Salazar-Gonzalez et al., 2008), 2 mM $MgSO_4$ and 0.2 mM dNTPs at 94°C for 2 min; (94°C for 15 s, 58.5°C for 30 s, and 68°C for 3 min) for 35 cycles; then 68°C for 15 min. Due to a primer mismatch, the first round of *env* amplification (Env1 PCR) for individual 605 was run in a 20 μL reaction volume with 0.5 U of Platinum Taq DNA Polymerase High Fidelity (Fisher Scientific, cat. 11-304-029) and outer PCR primers B3F3 (5′-TGGAAAGGTGAAGGGGCAGT-AGTAATAC-3′) (Salazar-Gonzalez et al., 2009) and Env3out (5′-TTGCTACTTGTGAT TGCTCCATGT-3′) (0.2 μM each), 2 mM $MgSO_4$ and 0.2 mM dNTPs at 94°C for 2 min; (94°C for 15 s, 60.4°C for 30 s, and 68°C for 6 min) for 40 cycles; then 68°C for 15 min.

The second round of *env* amplification (Env2 PCR) for all individuals was run in a 20 μL reaction volume with 1 μL of Env1 PCR product, 0.5 U of Platinum Taq DNA Polymerase High Fidelity (Fisher Scientific, cat. 11-304-029) and inner PCR primers Env5in (5′-TTAGGCATCTCCTATGGCAGGAAGAAG-3′) and Env3in (5′-GTCTCGAGATACTGCTCCCACCC-3′) (0.2 μM each) (Salazar-Gonzalez et al., 2008), 2 mM $MgSO_4$ and 0.2 mM dNTPs at 94°C for 2 min; (94°C for 15 s, 61°C for 30 s, and 68°C for 3 min) for 35 cycles; then 68°C for 15 min.

4 μL aliquots of Env2 PCR products were added to 16 μL aliquots of nuclease-free water and run on E-Gel 96 Agarose Gels with SYBR Safe DNA Gel Stain, 1% (ThermoFisher, cat. G720841) for visualization. Samples with a band size of ~2.5 kb were selected for sequencing. For each sample, a serial dilution of gDNA was performed to achieve a PCR product band on agarose gel for <30% of wells.

For *env* gene sequencing for latent clone TCR identification in individual 605, a shortened nested PCR was performed to amplify a ~500 bp amplicon of the *env* gene in which the expanded intact latent clone differed from other proviruses that were enriched. The first round of *env* amplification (Env1short PCR) for individual 605 was run in a 20 μL reaction volume with 0.5 U of Platinum Taq DNA Polymerase High Fidelity (Fisher Scientific, cat. 11-304-029) and outer PCR primers Env 1,133 F1 (5′-GAGGGGAATTTTTCTACTG TAACAC-3′) and Env 1,956 R1 (5′- GTTCTGCGAATTTT-CAATTAAGGTG -3′) (0.2 μM each), 2 mM $MgSO_4$ and 0.2 mM dNTPs at 94°C for 2min; (94°C for 15 s, 56°C for 30 s, and 68°C for 1 min) for 35 cycles; and 68°C for 15 min. The second round of *env* amplification (Env2short PCR) for individual 605 was run in a 20 μL reaction volume with 1 μL of Env1short PCR product, 0.5 U of Platinum Taq DNA Polymerase High Fidelity (Fisher Scientific, cat. 11-304-029) and inner PCR primers Env 1,240 F2 (5′- ATCA CACTCCGATGC-AGAATAAAAC-3′) and Env 1,765 R2 (5′-TTAGGTATCT-TTCCACAGCCAGTAC-3′) (0.2 μM each), 2 mM $MgSO_4$ and 0.2 mM dNTPs at 94°C for 2 min; (94°C for 15 s, 58°C for 30 s, and 68°C for 1 min) for 35 cycles; and 68°C for 15 min. Env2short PCR products were either sequenced by Sanger sequencing (GeneWiz, Azenta Life Sciences) with primers 1,240 F2 (5′-ATCACACTCCGATGC-AGAATAAAAC-3′) and Env 1,765 R2 (5′-TTAGGTATCT-TTCCACAGCCAGTAC-3′) or on the Illumina MiSeq platform as described below.

Env2 PCR product and NFL2 PCR product sequencing

DNA concentrations of second round PCR products were measured with Qubit dsDNA BR Assay Kit (ThermoFisher, cat. Q32853). Samples were diluted to a concentration of 10–20 ng/μL. For tagmentation, 1 μL of diluted second round PCR product was added to 0.25 μL Nextera TDE1 Tagment DNA enzyme and 1.25 μL of TD Tagmentation buffer (Illumina Tagment DNA TDE1 Enzyme and Buffer, Illumina cat. 20034198). Subsequently, DNA fragments were ligated to i5/i7 barcoded primers from the Illumina Nextera XT Index Kit v2 A – D (Nextera XT Index Kit v2 Set A, Illumina, cat. FC-131-2001 – FC-131-2004) using KAPA HiFi HotStart ReadyMix PCR Kit (Roche, cat. 07958935001). The ligated DNA fragments were pooled and purified with Agencourt RNAClean XP magnetic beads (Beckman Coulter, cat. A63987) for paired-end sequencing at a concentration of 12 pM with MiSeq Reagent Kit v2 (300-cycles) (Illumina, cat. MS-102-2002).

Sequences were assembled with the Defective and Intact HIV Genome Assembler.

Phylogenetic trees

Env sequences were aligned to the HIV HXB2CG *env* sequence using Geneious Prime software (version 11.0.12, Biomatters). Maximum-likelihood phylogenetic trees were built with PHYML, substitution model HKY85, without bootstrapping, to identify identical *env* sequences by clustering.

10× genomics

10x Genomics gene expression and V(D)J libraries were generated with the Chromium Single Cell 5′ Library & Gel Bead Kit (10x Genomics, cat. PN-1000014) and Chromium Single Cell V(D)J Enrichment Kit, Human T Cell (10x Genomics, cat. PN-1000005) as

described in the 10x Genomics protocol. The 5' expression library was sequenced with NovaSeq 6000 S1 (100 cycles) (Illumina, cat. 20012865) and the V(D)J library was sequenced with NextSeq 500/550 Mid Output Kit v2.5 (300 Cycles) (Illumina, cat. 20024905).

Latent clone TCR identification

Quiescent latent cells were enriched based on CD45RA, TRBC, and TRBV, and 5 cells per well were sorted into 96-well plates containing TCL buffer (Qiagen, cat. 1031576) with 1% beta-mercaptethanol and snap frozen on dry ice. Plates were stored at - 80°C until further use. After thawing on ice, magnetic bead clean-up was performed with Agencourt RNAClean XP magnetic beads (Beckman Coulter, cat. A63987). TCR mRNA was reverse transcribed into cDNA in a reaction volume of 20 μL with 200 U of SuperScript III Reverse Transcriptase (Invitrogen, cat. 18080044), 40 U of RNasin Plus Ribonuclease Inhibitor (Promega, cat. N2615), 0.5 mM dNTPs, 5 mM DTT, and the following primers: AC1R (5′-ACACATCAGAATCCTTACTTTG-3′), BC1R (5′-CAGTATCTGGAGT CATTGA-3′) (0.125 μM each) (Mamedov et al., 2013). After a second magnetic bead clean-up, binding TCR cDNA and gDNA, the first round of *env* amplification PCR from gDNA was performed as described above. After this, each well contained multiple copies of TCR cDNA and, if a latent cell was present, multiple copies of the *env* gene. A 1 μL aliquot from each well was taken for the second round of *env* amplification and sequencing to identify wells that contained a latent cell of the clone of interest (Env$^+$ wells). From all Env$^+$ wells and a random selection of Env$^-$ wells, an aliquot was taken to amplify the TCRα and TCRβ chain separately in duplicates with primers as previously described (Han et al., 2014). The first round of PCR amplification was performed with 2 μL aliquots of cDNA in a total reaction volume of 25 μL, using 0.75 U of HotStarTaq DNA polymerase (Qiagen, cat. 203209), the forward primers TRAV Ph1 (0.06 μM each) and the reverse primer AlphaPhase1 (0.03 μM) for TCRα amplification; the forward primers TRBV Ph1 (0.06 μM each) and the reverse primer BetaPhase1 (0.03 μM) for TCRβ amplification, and 0.2 mM dNTPs at 95°C for 15 min; (94°C for 30 s, 62°C for 1 min, and 72°C for 1 min) for 25 cycles; and 72°C for 5 min. The second round of PCR amplification was performed with 1 μL aliquots of first round PCR amplification products in a total reaction volume of 25 μL, using 0.75 U of HotStarTaq DNA polymerase (Qiagen, cat. 203209), the forward primers hTRAV (0.06 μM each) and the reverse primer AlphaPhase2 (0.03 μM) for TCRα amplification, the forward primers hTRBV (each at 0.06 μM) and the reverse primer BetaPhase2 (0.03 μM) for TCRβ amplification, and 0.2 mM dNTPs at 95°C for 15 min; (94°C for 30 s, 64°C for 1 min, and 72°C for 1 min) for 25 cycles; and 72°C for 5 min. The third round of PCR amplification was performed with 1 μL aliquots of second round PCR amplification products in a total reaction volume of 25 μL, using 0.75 U of HotStarTaq DNA polymerase (Qiagen, cat. 203,209), the AlphaBC primers (0.05 μM) for TCRα amplification, the BetaBC primers (0.05 μM) for TCRβ amplification, PlateNN primers (0.05 μM), PE1 and PE2 primers (0.05 μM each), and 0.2 mM dNTPs at 95°C for 15 min; (94°C for 30 s, 64°C for 1 min, and 72°C for 1 min) for 36 cycles; and 72°C for 5 min. The barcoded PCR amplification products were pooled, concentrated with Agencourt RNAClean XP magnetic beads (Beckman Coulter, cat. A63987), and run on an 1.5% agarose gel. The band between 300 and 400 bp was excised and purified with the NucleoSpin Gel and PCR Clean-up kit (Quiagen, cat. 740609.50). The purified product was subjected to paired-end sequencing at a concentration of 20 pM with MiSeq Reagent Kit v3 (600 cycle) (Illumina, cat. MS-102-3003).

PhiX-derived reads were removed from downstream analysis using a k-mer based approach implemented by bbduk.sh from BBTools v38.72 (https://sourceforge.net/projects/bbmap/). Samples were demultiplexed according to the combination of oligos used to uniquely identify the plate, row, and column they were placed (Han et al., 2014). The quality control check was performed with Trim Galore package v0.6.7 (https://github.com/FelixKrueger/TrimGalore) to trim Illumina adapters and low-quality bases. Paired-overlapping reads were exported into a single read by BBMerge. Subsequently, TRUST4 (Song et al., 2021) was used to reconstruct and annotate the T cell receptor (TCR) sequences. There is a chance of incorrect TCR assignment if the sequencing errors occur at the barcodes present in reads. A threshold was established based on the number of reads used to assemble a specific TCR contig assigned to an empty well. In most empty wells, less than 20 reads for a single TCR chain sequence were detected. An individual threshold was established for each sequencing run. We obtained the putative clonotypes, defined by the 10x Genomics V(D)J single-cell sequencing, that shared either the α or β chain found in that well. The most frequent clonotype associated with Env$^+$ wells in each individual was selected as the clonotype associated with proviral integration.

The bottleneck in this method is the *env* amplification from one single copy. The efficiency of the TCR amplification is documented as 88% for TCR α and 93% for TCR β based on published literature (Han et al., 2014). This is because TCR is abundantly expressed in T cells. Since either a unique TCR α or β chain is sufficient to identify a TCR of interest from the 10× TCR sequencing data, the detection rate for the specific TCR in Env$^+$ wells is rather high.

QUANTIFICATION AND STATISTICAL ANALYSIS

Single-cell RNA-seq and single-cell TCR-seq processing

Single-cell RNA-seq binary base call (BCL) files were demultiplexed and converted into FASTQ files using BCLtoFastq prior to alignment to hg38 with CellRanger (v4.0.0) and analyzed in R studio with Seurat (v4). Cells with a mitochondrial proportion greater than 5% and/or a feature count <200 or >2,500 were discarded. Sample batches were combined, normalized and scaled with SCTransform. Uniform Manifold Approximation and Projection (UMAP) clustering was performed selecting the first thirty principal components. Single-cell TCR-seq FASTQs were aligned with CellRanger (v4.0.0) to the default CellRanger VDJ reference. Output contig annotations were filtered and analyzed in R studio with Seurat.

Mapping scRNA-seq to CD4$^+$ T cells reference

CD4$^+$ T cell population was extracted from published human peripheral blood cells multimodal annotated reference (Hao et al., 2021). The UMAP reference from extracted CD4$^+$ T population was recreated using the first 50 principal components of the RNA expression slot and the cells from each individual were anchored and mapped utilizing the FindTransferAnchors and MapQuery functions from Seurat (reference.reduction = "pca", dims = 1:50, reduction.model = umap).

HIV-1 transcript detection using 10× data

Samtools was used to extract reads that cellranger failed to align to the human reference. Sequences extracted from the BAM file generated by cellranger contain identified cell barcodes and UMI in the sequence header. We used bbduk.sh from the BBtools package to search for reads containing HIV-1 k-mers (k = 31) from intact HIV-1 genome sequences obtained from the Los Alamos HIV database. Cell barcode and UMI were extracted from sequences containing HIV-1 k-mers and used to calculate HIV-1 expression.

Cell Reports, Volume *40*

Supplemental information

Distinct gene expression by expanded clones of quiescent memory $CD4^+$ T cells harboring intact latent HIV-1 proviruses

Georg H.J. Weymar, Yotam Bar-On, Thiago Y. Oliveira, Christian Gaebler, Victor Ramos, Harald Hartweger, Gaëlle Breton, Marina Caskey, Lillian B. Cohn, Mila Jankovic, and Michel C. Nussenzweig

ID	Age	Sex	Race	Year HIV-1 Dx	Year ART initiation	Uninterr. ART (years)	Viral load (copies/mL)	$CD4^+$ T cell count	Reported nadir	ART regimen
**B207**	47	M	White/Hispanic	10	10	9	<20	724	50	EFV/TDF/FTC
**603**	43	M	White/Hispanic	12	10	10	<20	300	693	EFV/TDF/FTC
**605**	36	M	White/Hispanic	15	14	2	<20	372	524	RPV/TDF/FTC
**5104**	35	M	Black	7	7	7	<20	400	606	BIC/TAF/FTC
**5125**	34	M	Black	11	10	2	<20	450	1006	DTG/TDF/FTC
**9247**	31	M	Black	6	6	6	<20	728	600	EVG/Cobi/TAF/FTC

Table S1: Clinical characteristics of study participants. Related to STAR methods – Participant cohort.
Dx: diagnosis; Uninterr: uninterrupted; ART: antiretroviral treatment; EVG: elvitegravir; Cobi: cobicistat; TDF: tenofovir disoproxil fumarate; FTC: emtricitabine; RPV: rilpivirine; TAF: tenofovir alafenamide fumurate; BIC: bictegravir.

	603	605	B207	5104	5125	9247
**CUPM Q4PCR**	ND	ND	ND	210[1]	28[1]	15[2]
**CUPM Env PCR**	13	431	164	ND	ND	ND
**Latent clone inducible *in vitro***	Yes[3]	Yes[3]	Yes[3,4]	Yes[4]	ND	No
**Integration site**	ZNF486[4]	ND	ZPF30[4]	ATP2B4, DUXL6[4]	ND	ND

Table S2: Frequency, inducibility, and integration site of the clone of interest in CD4⁺ T cells. Related to STAR methods – Participant cohort.
CUPM: Clonal units of clone of interest per million CD4⁺ T cells.
[1]Gaebler (2022) [2]Gaebler (2021) [3]Cohn (2018) [4]Huang (2021)

	CD45RA	TRBC	TRBV	combined
**603**	1.5	2.2	14.3	47.2
**605**	~2 (estimate)	-	20	40
**B207**	1.6	2	2.9	9.3
**5104**	2.4	1.6	2.9	11.1
**5125**	1.5	1.8	20	54
**9247**	1.9	5	71	674.5

Table S3: Relative enrichment per marker based on flow cytometry. Related to Figure 3 and Figure S2.

	Latent clone *env* copies per 10^4 CD4⁺ T cells	Specific TCR per 10^4 CD4⁺ T cells	Ratio TCR/*env*
**603**	20	45	2.3
**605**	163	502	3.1
**B207**	6	14	2.3
**5104**	14	16	1.1
**5125**	15	17	1.1
**9247**	18	23	1.3

Table S4: Comparison between *env* copies per 10^4 CD4⁺ T cells (based on Env PCR) and the frequency of the latent clone TCR (based on 10x Genomics TCR sequencing) after enrichment based on CD45RA, TRBV, and TRBC. Related to Figure 3 and Figure 4.

	**5104**		
	**10x TCR clonotype**	**Env^{+} (n=7)**	**Env^{-} (n=36)**
1	clonotype10133	100.0	0.0
2	clonotype66526	28.6	33.3
3	clonotype1969	14.3	11.1
4	clonotype66656	14.3	8.3
5	clonotype80542	14.3	13.9
6	clonotype19143	0.0	5.6
	**5125**		
	**clonotype**	**Env^{+} (n=4)**	**Env^{-} (n=31)**
1	clonotype91937	100.0	0.0
2	clonotype11134	25.0	3.3
3	clonotype44083	0.0	3.3
4	clonotype64266	0.0	3.3
5	clonotype89427	0.0	3.3
6	clonotype10571	0.0	0.0
	**9247**		
	**clonotype**	**Env^{+} (n=9)**	**Env^{-} (n=42)**
1	clonotype87647	88.9	0.0
3	clonotype5053	0.0	3.0
4	clonotype28375	0.0	24.2
5	clonotype72048	11.1	21.2
6	clonotype62633	11.1	18.2
7	clonotype62638	11.1	18.2
8	clonotype27093	11.1	0.0
9	clonotype46171	11.1	6.1
10	clonotype556	11.1	0.0
11	clonotype93203	11.1	0.0

Table S5: Identification of the latent clone TCR in individuals 5104, 5125, and 9247. Related to Figure 4.
Frequency of the most frequent 10x TCR clonotypes in Env^{+} and Env^{-} wells for individuals 5104, 5125, and 9247 shows a clear enrichment of only one TCR clonotype in Env^{+} wells. This TCR clonotype was designated as the latent clone TCR.

Number of cells analyzed by 10x genomics single cell gene expression							
**Individual**	**603**	**605**	**B207**	**5104**	**5125**	**9247**	**Sum**
**# of cells**	16,224	17,357	27,472	21,143	12,173	14,848	109,217

**Individual**	**603**			**605**			**B207**			**5104**			**5125**			**9247**		
**Clone**	smaller (n=64)	latent (n=75)	bigger (n=84)	smaller (n=330)	latent (n=844)	bigger	smaller (n=40)	latent (n=41)	bigger (n=42)	smaller (n=32)	latent (n=35)	bigger (n=38)	smaller (n=21)	latent (n=21)	bigger (n=22)	smaller (n=34)	latent (n=34)	bigger (n=35)
**Cluster 0**	17.2	18.7	34.5	8.5	7.8	-	0.0	12.2	35.7	43.8	17.1	0.0	33.3	9.5	9.1	8.8	0.0	0.0
**Cluster 1**	54.7	0.0	4.8	68.2	2.7	-	5.0	0.0	26.2	0.0	5.7	0.0	19.0	14.3	36.4	2.9	2.9	5.7
**Cluster 2**	1.6	0.0	0.0	0.0	0.0	-	0.0	0.0	11.9	40.6	8.6	0.0	28.6	4.8	27.3	20.6	23.5	34.3
**Cluster 3**	1.6	0.0	0.0	1.5	0.6	-	5.0	4.9	4.8	3.1	0.0	0.0	9.5	0.0	9.1	0.0	0.0	0.0
**Cluster 4**	3.1	2.7	7.1	4.5	1.2	-	25.0	2.4	0.0	3.1	2.9	0.0	0.0	4.8	13.6	35.3	8.8	31.4
**Cluster 5**	4.7	10.7	10.7	11.5	1.7	-	0.0	0.0	4.8	6.3	0.0	0.0	4.8	4.8	0.0	5.9	0.0	5.7
**Cluster 6**	4.7	0.0	0.0	0.0	0.7	-	0.0	0.0	2.4	0.0	0.0	0.0	4.8	0.0	4.5	23.5	35.3	22.9
**Cluster 7**	0.0	60.0	23.8	2.1	63.7	-	0.0	73.2	4.8	3.1	65.7	0.0	0.0	47.6	0.0	0.0	29.4	0.0
**Cluster 8**	0.0	8.0	0.0	0.0	3.8	-	0.0	7.3	0.0	0.0	0.0	100.0	0.0	9.5	0.0	0.0	0.0	0.0
**Cluster 9**	0.0	0.0	0.0	0.0	0.5	-	2.5	0.0	0.0	0.0	0.0	0.0	0.0	0.0	0.0	0.0	0.0	0.0
**Cluster 10**	0.0	0.0	0.0	0.3	0.2	-	62.5	0.0	0.0	0.0	0.0	0.0	0.0	0.0	0.0	0.0	0.0	0.0
**Cluster 11**	7.8	0.0	16.7	1.8	16.1	-	0.0	0.0	9.5	0.0	0.0	0.0	0.0	4.8	0.0	0.0	0.0	0.0
**Cluster 12**	0.0	0.0	2.4	0.0	0.4	-	0.0	0.0	0.0	0.0	0.0	0.0	0.0	0.0	0.0	2.9	0.0	0.0
**Cluster 13**	4.7	0.0	0.0	0.6	0.2	-	0.0	0.0	0.0	0.0	0.0	0.0	0.0	0.0	0.0	0.0	0.0	0.0
**Cluster 14**	0.0	0.0	0.0	0.9	0.4	-	0.0	0.0	0.0	0.0	0.0	0.0	0.0	0.0	0.0	0.0	0.0	0.0
**Sum**	100	100	100	100	100	-	100	100	100	100	100	100	100	100	100	100	100	100

Table S6: Upper part: Number of cells analyzed by 10x Genomics per individual. Lower part: Distribution [%] of the latent clone, the next smaller, and the next bigger clone per individual over the 15 gene expression clusters. Related to Figure 5.

Individual	Cluster 7					
	# of cells	# of latent cells	# [%] of other cells	# [%] of other cells in clones	# of other clones	# [%] of singlet T cells
603	887	45	842 [94.9]	581 [65.5]	330	261 [29.4]
605	1,989	538	1,451 [73]	1,156 [58.1]	498	295 [14.8]
B207	1,295	30	1,265 [97.7]	890 [68.7]	526	375 [29]
5104	960	23	937 [97.6]	655 [68.2]	383	282 [29.4]
5125	297	10	287 [96.6]	223 [75.1]	111	64 [21.5]
9247	358	10	348 [97.2]	318 [88.8]	100	30 [8.4]

Table S7: Contribution of non-latent clones and single cells to cluster 7. Related to Figure 5.

Ensembl gene ID	External gene name	p value	Average log2 fold change
ENSG00000115687	PASK	2.5074E-273	-0.937004582
ENSG00000188404	SELL	1.1432E-303	-0.757375402
ENSG00000168209	DDIT4	4.2236E-135	-0.753863566
ENSG00000070756	PABPC1	1.3615E-275	-0.577338107
ENSG00000074800	ENO1	3.9911E-72	-0.552495574
ENSG00000273149	AL138963.4	1.69448E-43	-0.503468146
ENSG00000111669	TPI1	3.75841E-56	-0.502939955
ENSG00000095794	CREM	7.5629E-48	-0.48733596
ENSG00000157601	MX1	3.44639E-38	-0.463685933
ENSG00000149212	SESN3	2.09806E-96	-0.458160858
ENSG00000126353	CCR7	5.6185E-113	-0.438022627
ENSG00000177410	ZFAS1	9.1716E-145	-0.432744809
ENSG00000173762	CD7	1.2733E-119	-0.422179842
ENSG00000059804	SLC2A3	4.68348E-68	-0.401753273
ENSG00000197061	HIST1H4C	1.74666E-54	-0.367052221
ENSG00000234741	GAS5	1.8049E-124	-0.365481063
ENSG00000134333	LDHA	3.62429E-18	-0.356852098
ENSG00000197989	SNHG12	2.6524E-59	-0.354230806
ENSG00000105220	GPI	2.30295E-38	-0.33651571
ENSG00000146278	PNRC1	2.56242E-66	-0.33232545
ENSG00000196352	CD55	3.58249E-86	-0.331613182
ENSG00000269028	MTRNR2L12	9.28887E-42	-0.326076812
ENSG00000251562	MALAT1	4.23243E-61	-0.326073295
ENSG00000240972	MIF	1.16951E-48	-0.325935464
ENSG00000067225	PKM	3.0003E-53	-0.324450491
ENSG00000166012	TAF1D	4.1997E-62	-0.323635813
ENSG00000130066	SAT1	6.88644E-24	-0.320711104
ENSG00000161011	SQSTM1	3.25091E-23	-0.307785538
ENSG00000181163	NPM1	1.7052E-145	-0.295019474
ENSG00000081059	TCF7	2.53957E-59	-0.293559954
ENSG00000100906	NFKBIA	6.90414E-22	-0.286544704
ENSG00000138795	LEF1	4.65739E-63	-0.283635236
ENSG00000102144	PGK1	3.47758E-18	-0.283588302
ENSG00000104765	BNIP3L	4.68824E-42	-0.280401542
ENSG00000144381	HSPD1	1.33312E-51	-0.27754675
ENSG00000096384	HSP90AB1	6.19504E-51	-0.27513593
ENSG00000114023	FAM162A	3.98958E-38	-0.271026449
ENSG00000167658	EEF2	2.0542E-116	-0.263184273
ENSG00000081320	STK17B	1.01417E-40	-0.263109056
ENSG00000105193	RPS16	7.12703E-82	-0.25357902

ENSG00000213145	CRIP1	1.84831E-74	0.251381711
ENSG00000204642	HLA-F	1.67539E-32	0.252987681
ENSG00000064666	CNN2	7.14478E-34	0.254603703
ENSG00000136167	LCP1	4.88255E-38	0.255115632
ENSG00000145247	OCIAD2	1.35036E-33	0.255136956
ENSG00000135441	BLOC1S1	8.55057E-29	0.256708541
ENSG00000217555	CKLF	6.26258E-37	0.268552743
ENSG00000179218	CALR	5.15079E-26	0.269118004
ENSG00000166710	B2M	0	0.270596986
ENSG00000034713	GABARAPL2	7.97742E-47	0.271789708
ENSG00000213626	LBH	1.70427E-35	0.275234928
ENSG00000158062	UBXN11	2.96782E-31	0.277861974
ENSG00000135046	ANXA1	6.7754E-104	0.277916253
ENSG00000240065	PSMB9	9.1741E-63	0.278633877
ENSG00000165929	TC2N	3.38931E-34	0.282431558
ENSG00000136810	TXN	4.66479E-33	0.284410494
ENSG00000115232	ITGA4	4.07453E-43	0.296034223
ENSG00000234745	HLA-B	2.442E-301	0.299434559
ENSG00000182718	ANXA2	1.2129E-48	0.300086815
ENSG00000105404	RABAC1	3.41798E-59	0.300856113
ENSG00000108518	PFN1	5.1347E-192	0.308425928
ENSG00000075624	ACTB	3.1245E-173	0.312823868
ENSG00000163191	S100A11	5.8168E-123	0.313585328
ENSG00000213719	CLIC1	3.2501E-80	0.321146717
ENSG00000126246	IGFLR1	1.12795E-32	0.321234367
ENSG00000027869	SH2D2A	1.2519E-59	0.325040074
ENSG00000008517	IL32	1.885E-203	0.326877861
ENSG00000100300	TSPO	4.23815E-58	0.328869744
ENSG00000130592	LSP1	5.13406E-85	0.335795569
ENSG00000170571	EMB	1.30243E-48	0.340520299
ENSG00000110324	IL10RA	7.30307E-71	0.345878196
ENSG00000092841	MYL6	1.8186E-212	0.349021829
ENSG00000160255	ITGB2	1.63101E-55	0.376681806
ENSG00000197747	S100A10	5.2906E-220	0.381136641
ENSG00000169442	CD52	2.9441E-231	0.386540002
ENSG00000132965	ALOX5AP	2.30411E-89	0.390330172
ENSG00000148362	PAXX	1.9717E-94	0.391613282
ENSG00000197540	GZMM	5.469E-80	0.411312119
ENSG00000002586	CD99	1.1703E-191	0.411377898
ENSG00000111796	KLRB1	8.73173E-56	0.434616046
ENSG00000196154	S100A4	9.403E-205	0.446707794

ENSG00000142669	SH3BGRL3	0	0.473220735
ENSG00000126264	HCST	3.4505E-135	0.484457968
ENSG00000103187	COTL1	8.1033E-136	0.491151274
ENSG00000100097	LGALS1	3.0001E-118	0.520481509
ENSG00000116824	CD2	7.0921E-219	0.527921045
ENSG00000051523	CYBA	0	0.535424541
ENSG00000133321	PLAAT4	1.0403E-293	0.6130956
ENSG00000235576	LINC01871	6.8684E-252	0.712737658
ENSG00000186810	CXCR3	0	0.863806843
ENSG00000145220	LYAR	0	0.91690397
ENSG00000223865	HLA-DPB1	0	1.005834612
ENSG00000077984	CST7	0	1.00668931
ENSG00000231389	HLA-DPA1	0	1.0517126
ENSG00000145649	GZMA	0	1.159266639
ENSG00000196126	HLA-DRB1	0	1.189048765
ENSG00000158050	DUSP2	0	1.377812382
ENSG00000271503	CCL5	0	1.450021207
ENSG00000019582	CD74	0	1.478166065
ENSG00000113088	GZMK	0	2.055910352

Table S8: Differentially expressed genes (average log2fold < -2.5; average log2fold > 2.5) in cluster 7 compared to all other clusters. Related to Figure 6.

	**603**	**605**	**B207**	**5104**	**5125**	**9247**
**CD4 CTL**	1.6%	58.5%	0.0%	0.0%	0.9%	0.0%
**CD4 Naïve**	0.0%	1.0%	0.0%	0.0%	0.0%	0.0%
**CD4 Proliferating**	0.0%	0.0%	0.0%	0.0%	0.0%	0.0%
**CD4 TCM**	0.1%	2.2%	0.0%	0.1%	0.1%	0.2%
**CD4 TEM**	6.1%	21.9%	2.4%	1.1%	3.8%	2.0%
**Treg**	0.0%	0.4%	0.0%	0.0%	0.0%	0.0%

Table S9: Percentage of cell pertaining to the latent clone relative to cluster size. Related to Figure 7.

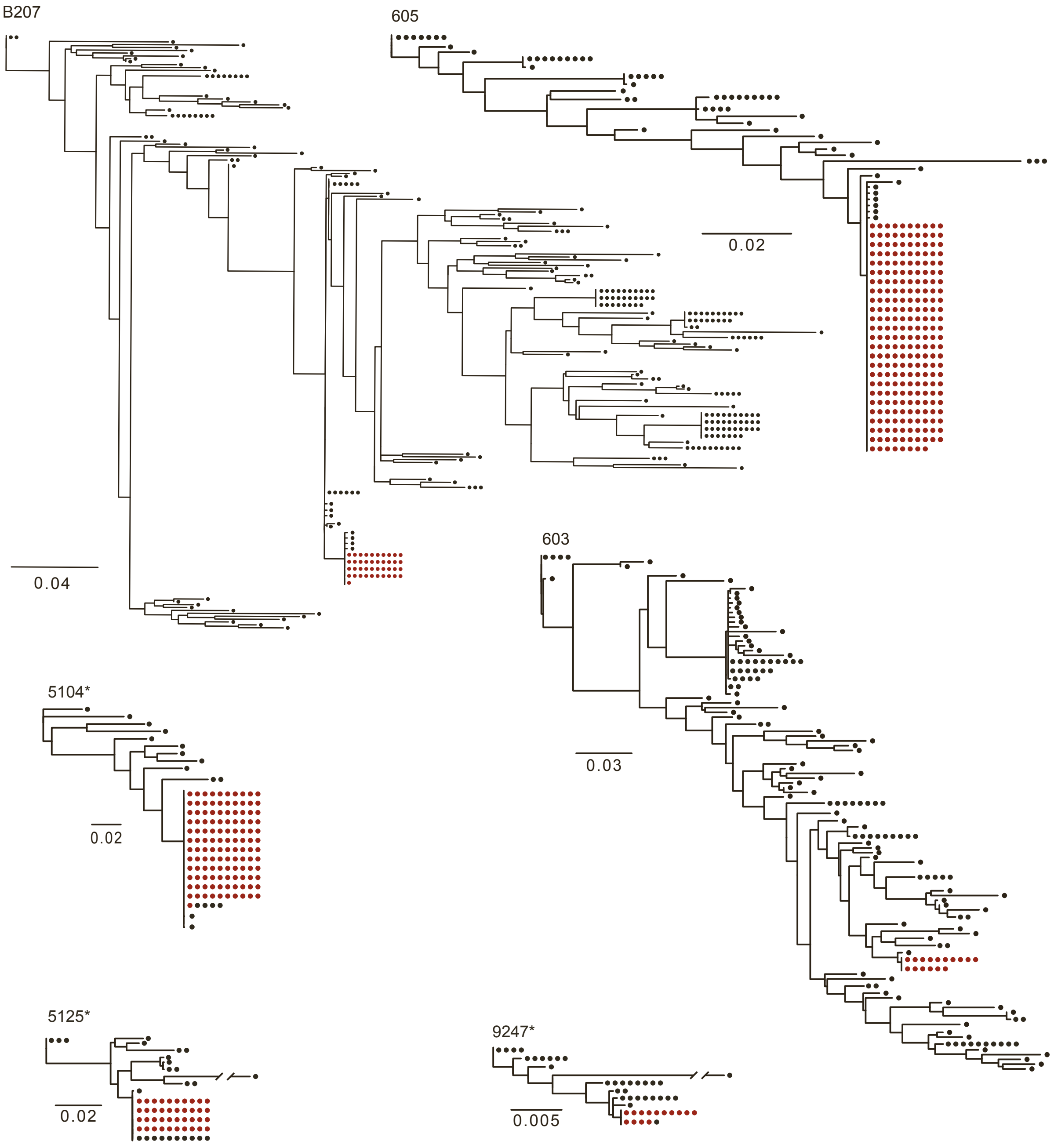

Figure S1 The latent reservoir in each individual contains an expanded intact latent clone. Related to STAR methods - Participant cohort.

Maximum-likelihood phylogenetic trees show the *env* gene of the clone of interest marked in red. The scale bars indicate the number of substitutions per site. Each dot on the maximum-likelihood phylogenetic trees represents an *env* sequence that was recovered by Env PCR (individuals B207, 603, and 605) or Q4PCR (marked with asterisk; individuals 5104, 5125, and 9247 (Gaebler et al., 2021; Gaebler et al., 2022)) from CD4^{+} T cells before enrichment. For individuals 603, 605, and B207 the *env* gene was also found in viral outgrowth assays (Cohn et al., 2018).

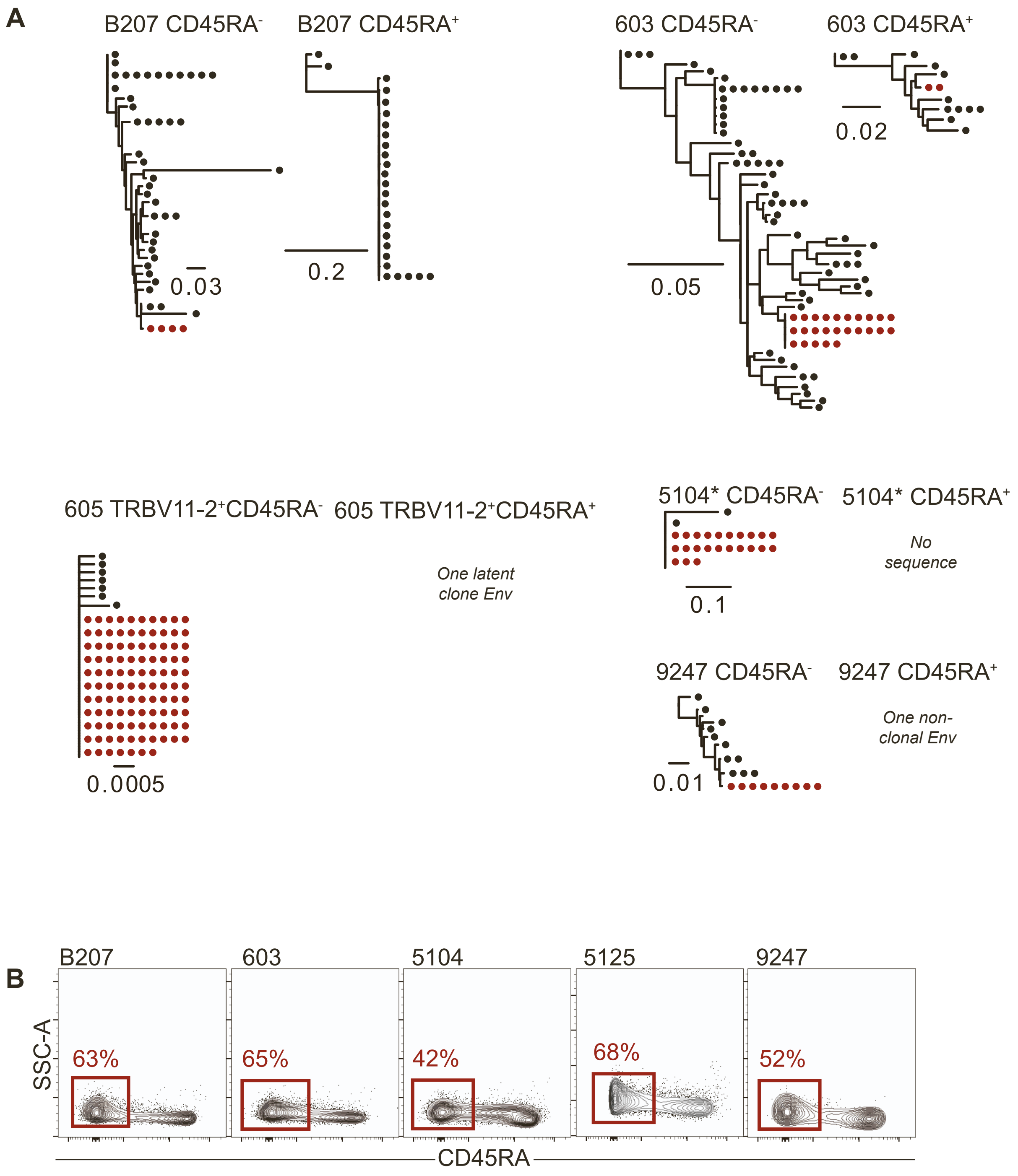

Figure S2 The latent clone of interest is enriched in the CD45RA$^-$ compartment. Related to Figure 1 and Table S3.

Maximum-likelihood phylogenetic trees show *env* gene of the clone of interest marked in red. The scale bars indicate the number of substitutions per site. Asterisks indicate trees based on *env* sequences obtained from Q4PCR (Gaebler et al., 2019), all other trees are based on Env PCR. Each sort was performed once.

(A) Magnetic negative selection of CD45RA$^-$ memory CD4$^+$ T cells enriches the latent clone of interest in all 6 individuals.

(B) Flow cytometry plots indicating the fraction of CD45RA$^-$ cells among CD4$^+$ T cells.

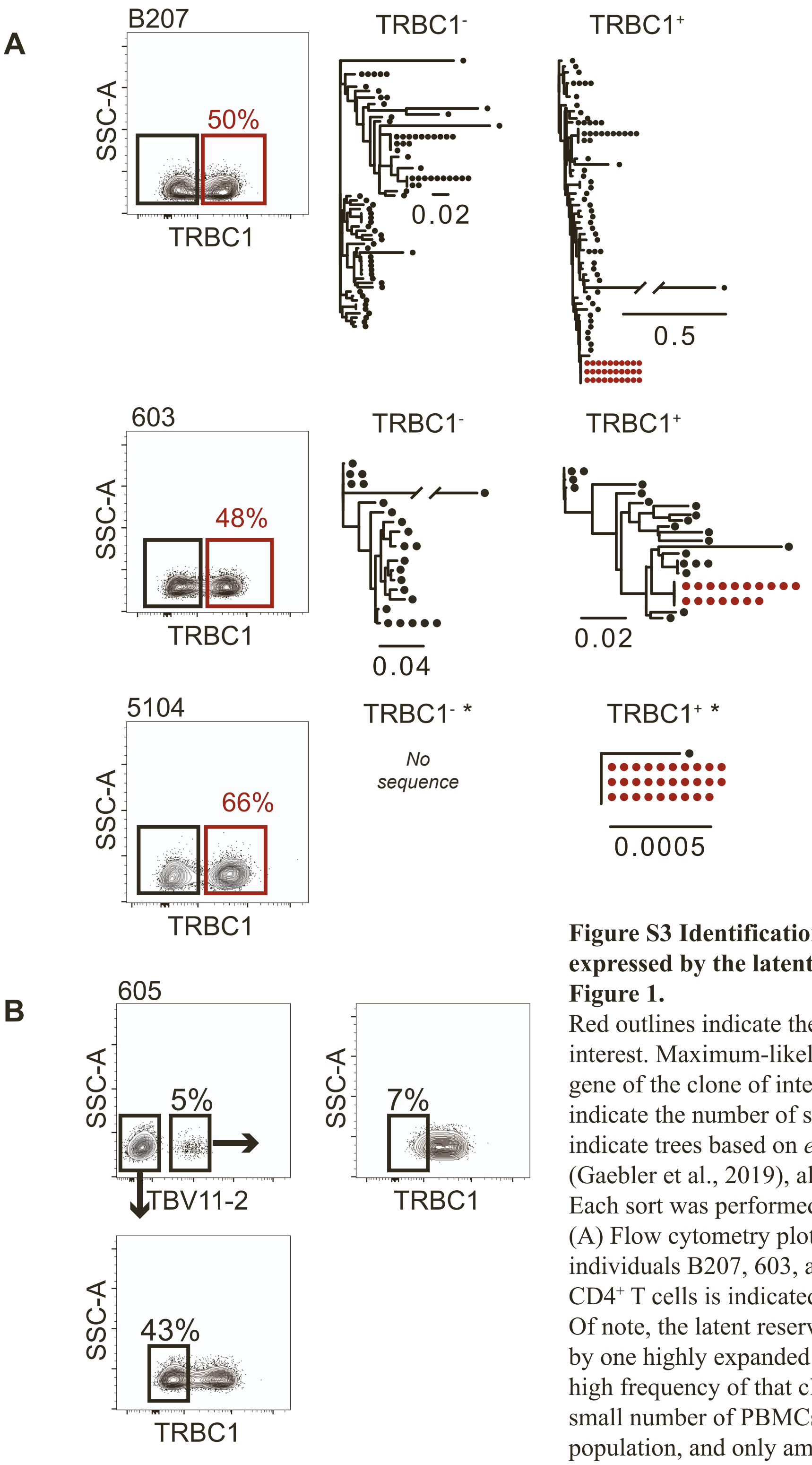

Figure S3 Identification of the TRBC domain that is expressed by the latent clone of interest. Related to Figure 1.
Red outlines indicate the population containing the clone of interest. Maximum-likelihood phylogenetic trees show *env* gene of the clone of interest marked in red. The scale bars indicate the number of substitutions per site. Asterisks indicate trees based on *env* sequences obtained from Q4PCR (Gaebler et al., 2019), all other trees are based on Env PCR. Each sort was performed once.
(A) Flow cytometry plots show TRBC1 staining for individuals B207, 603, and 5104. The fraction of TRBC1$^+$ CD4$^+$ T cells is indicated.
Of note, the latent reservoir of individual 5104 is dominated by one highly expanded single clone (Figure S1). Due to the high frequency of that clone, we only needed to assay a small number of PBMCs to detect that clone in the TRBC1$^+$ population, and only amplified one other sequence by Q4PCR. Therefore, much less frequent latent cells that possibly were TRBC1$^-$ were not captured.
(B) Flow cytometry plots show TRBC1 staining for TRBV11-2$^-$ and TRBV11-2$^+$ cells in individual 605. The fraction cells within a gate is indicated above the gate.
